# Burden of Shigella and enterotoxigenic Escherichia coli infections among children under 5 years in Ethiopia, Kenya and Malawi: a systematic review and meta-analysis

**DOI:** 10.1136/bmjgh-2024-018515

**Published:** 2026-03-02

**Authors:** Chikondi Andrew Mwendera, Mengistu Yilma, Celestine Wairimu, Kelvin Kering, Edson Mwinjiwa, James Ngumo Kariuki, Daniel Asrat, Amha Mekasha, Chisomo Msefula, Samuel Kariuki, Jen Cornick, Helen Clough, Neil French, Virginia Pitzer, Khuzwayo C Jere, Daniel Hungerford, GHRG-GI Consortium, Catherine Beavis

**Affiliations:** 1 Clinical Infection, Microbiology and Immunology, University of Liverpool Institute of Infection Veterinary and Ecological Sciences, Liverpool, UK; 2 Addis Ababa University College of Health Sciences, Addis Ababa, Oromia, Ethiopia; 3 KEMRI, Nairobi, Nairobi, Kenya; 4 Malawi-Liverpool-Wellcome Trust Clinical Research Programme, Blantyre, Blantyre, Malawi; 5 Kenya Medical Research Institute, Nairobi, Nairobi County, Kenya; 6 Pathology, Kamuzu University of Health Sciences, Blantyre, Malawi; 7 National Institute for Health and Care Research Health Protection Research Unit in Gastrointestinal Infections, University of Liverpool, Liverpool, UK; 8 Department of Epidemiology of Microbial Diseases, Yale University, New Haven, Connecticut, USA; 9 Department of Clinical Infection, Microbiology and Immunology, University of Liverpool Institute of Infection Veterinary and Ecological Sciences, Liverpool, Merseyside, UK; 10 Clinical Infection, Microbiology and Immunology, University of Liverpool, Liverpool, Merseyside, UK

**Keywords:** Systematic review, Bacillary dysentery, Child health, Global Health, Africa South of the Sahara

## Abstract

Diarrhoea remains a major problem among children in low- and middle-income countries, driven by multiple pathogens including rotavirus, *Shigella* and enterotoxigenic *Escherichia coli* (ETEC). Rotavirus vaccines have notably reduced diarrhoea deaths. However, the health consequences associated with *Shigella* and ETEC, along with rising antimicrobial resistance (AMR), have prompted the WHO to prioritise vaccine development against these two pathogens. Understanding their disease burden is crucial for guiding this effort and informing preparedness for vaccine adoption.

We conducted a systematic review and meta-analysis of primary peer-reviewed literature to establish the prevalence, subtypes and AMR patterns of *Shigella* and ETEC-associated diarrhoea in Ethiopia, Kenya and Malawi, where the authors have established a multidisciplinary research programme addressing gastrointestinal infections. We searched in PubMed, among other databases, for English-language publications from 1 January 2000 to 28 July 2023. The meta-analysis used a random effects model to estimate pooled prevalence.

43 studies were included. Malawi exhibited a higher estimated prevalence of *Shigella* (24% (95% CI 10% to 39%)) than Ethiopia and Kenya (both with an estimated prevalence of 6%), most likely explained by the application of sensitive, molecular detection methods in Malawi. The overall pooled prevalence of *Shigella* was 8% (95% CI 6% to 9%). Malawi again displayed higher ETEC prevalence (24% (95% CI 14% to 33%)) compared with Kenya (7% (95% CI 5% to 10%)), with no studies of ETEC identified from Ethiopia. The overall pooled prevalence of ETEC was 11% (95% CI 6% to 15%). *Shigella flexneri* was the major species of *Shigella,* and heat-stable ETEC was highly prevalent. *Shigella* species displayed resistance to several classes of antibiotics, including penicillins, tetracyclines, macrolides and sulphonamides, but susceptibility to fluoroquinolones and cephalosporins.

These findings underscore the need for countries to generate updated disease burden estimates for *Shigella* and ETEC through epidemiologically robust studies that use sensitive diagnostic methods in preparation for vaccine introduction.

**WHAT IS ALREADY KNOWN ON THIS TOPIC**

**WHAT THIS STUDY ADDS**

- This systematic review highlights a scarcity of focused research on these pathogens, particularly concerning ETEC within the study countries.
- The extent of diarrhoea attributable to *Shigella* and ETEC is significant in Malawi and likely underestimated in Ethiopia and Kenya, with the ETEC burden largely unknown in Ethiopia.

**HOW THIS STUDY MIGHT AFFECT RESEARCH, PRACTICE OR POLICY**

- LMICs should prioritise the use of standardised and sensitive PCR techniques in epidemiologically robust research studies and disease surveillance to accurately determine pathogen-specific diarrhoea among children. This approach can inform the development of effective interventions and the introduction of enteric vaccines.

## Introduction/background

 Globally, diarrhoea is the third leading cause of mortality among children under the age of 5 years, causing an estimated 1.7 billion childhood diarrhoea cases and more than 400 000 deaths each year.^1^ Despite a global decrease in childhood diarrhoeal mortality over the past two decades, the burden is still considerably higher in low- and middle-income countries (LMICs) than in high-income countries, particularly in sub-Saharan Africa (SSA).

Although the introduction of rotavirus vaccines has significantly reduced rotavirus-related diarrhoea, rotavirus remains the leading cause of diarrhoea among children.^2^ In addition, *Shigella* and enterotoxigenic *Escherichia coli* (ETEC) are responsible for up to 70% of childhood diarrhoeal cases caused by bacteria and are associated with moderate-to-severe diarrhoea in SSA.^3^

Despite the existence of lifesaving interventions for preventing *Shigella* and ETEC, such as improved sanitation, hygiene and access to safe drinking water, coverage and accessibility remain a challenge in LMICs.^4 5^ Therefore, antibiotic treatment is an essential component in their management. For *Shigella* infections, the WHO recommends ciprofloxacin as the first choice for treating children with *Shigella* dysentery, although antimicrobial resistance (AMR) to this antibiotic is increasing.^6^ There are also resistance concerns with second-line antibiotics, including azithromycin, cefixime and ceftriaxone, while trimethoprim-sulfamethoxazole, once considered an alternative, also faces widespread resistance.^6^ For ETEC infections, the WHO recommends fluoroquinolones and other antibiotics, including ampicillin, doxycycline and azithromycin, with prescriptions being country-dependent due to local resistance patterns.^7^ However, increased misuse of these drugs in LMICs has led to a rise in AMR among ETEC strains.^8^

Due to growing AMR concerns for *Shigella* and ETEC infections and the challenges of other prevention strategies, exploring alternative interventions, such as effective vaccines, has become a greater public health need. This has prompted the WHO to prioritise the development of vaccines against *Shigella* and ETEC.^9 10^

Accurately quantifying the disease burden of *Shigella* and ETEC in SSA countries is a WHO priority research area.^11^ This understanding is essential to justify the development of vaccines, to provide a basis for evaluation and to motivate demand from policymakers, especially in LMICs, to support the future incorporation of these vaccines into routine immunisation programmes.

The National Institute for Health and Care Research funded the establishment of the Global Health Research Group on Gastrointestinal Infections in Ethiopia, Kenya and Malawi. This

systematic review was conducted as an adjunct to prospective diarrhoea surveillance studies to quantify the burden of diarrhoeal diseases caused by *Shigella* and ETEC in these three countries in Eastern and Southern Africa.

## Materials and methods

This review was planned, conducted and reported in accordance with the guidelines for Preferred and Reporting Items for Systematic Reviews and Meta-analyses.^12^ The review protocol was published in the Prospective Register of Systematic Reviews (PROSPERO) (reference number: CRD42023438747) (an international registry for systematic review protocols aimed at promoting transparency, preventing duplication and reducing bias in evidence synthesis) and was created to assess the burden of rotavirus, *Shigella* and ETEC in Ethiopia, Kenya and Malawi. However, due to the extensive volume of literature, and rotavirus vaccination already being in use, here we focus on *Shigella* and ETEC. This approach has allowed for a more in-depth and comprehensive analysis of the data related to each pathogen, ensuring that the findings are thoroughly explored and presented in a detailed manner.

We developed a search strategy customised to retrieve recently published primary research (from 1 January 2000, when the Millennium Development Goals were developed, to 28 July 2023) from five databases: PubMed, Web of Science, Medical Literature Analysis and Retrieval System Online (MEDLINE) (Ovid), Cumulative Index to Nursing and Allied Health Literature (CINAHL) and the African Index Medicus (online supplemental appendix 1). The following keywords or alternative terms (detailed in the supplementary search strategy) were combined using Boolean operators:^13^ “children under the age of five years”, “Ethiopia”, “Kenya”, “Malawi”, “*Shigella*”, “Rotavirus”, “enterotoxigenic *Escherichia coli*” and “disease burden”. Furthermore, we manually conducted cross-referencing of the included studies’ references to identify additional relevant studies that might have been overlooked during the initial online searches.

### Study selection criteria

The following criteria were applied to include primary peer-reviewed studies: (1) focus on *Shigella* and ETEC; (2) children under the age of 5 years; (3) relevant outcome measures reported related to disease burden, including prevalence, incidence and morbidity—the studies included encompassed epidemiological, surveillance, clinical, ecological and observational designs, such as cross-sectional, cohort or case-control studies (data from the cases only were extracted); (4) conducted specifically in Ethiopia, Kenya and Malawi (multicountry studies including these countries were included, with only data from these countries extracted); (5) intervention studies (randomised controlled studies) assessing the effectiveness of various control and prevention strategies, such as vaccination, hygiene promotion and water and sanitation (data were extracted only from the control/placebo arm of these studies).

Studies were excluded that (1) focused exclusively on the cost-effectiveness of *Shigella* and ETEC; (2) did not produce empirical data; (3) were community studies conducted on asymptomatic children; (4) consisted of case reports, commentaries, review articles or editorials and/or (5) were not published in English.

### Screening process

The results obtained from the online database searches were uploaded into the Rayyan online platform, which facilitates the management of systematic reviews, including functions such as resolving duplicates and screening.^14^

A team of six reviewers (authors CMW, MY, JK, KK, CW and EM) was divided into three pairs, one for each country included in the review. Each pair independently screened a list of publications, initially evaluating titles and abstracts. Full papers of potentially relevant studies were then sought and further reviewed and screened by the team. Any conflicting decisions were discussed and resolved within each pair, with consultation from a third reviewer in cases where consensus was not achieved.

### Data extraction

A data extraction tool was developed and tested on a subset of randomly selected studies by each member of the review team (authors CMW, MY, JK, KK, CW and EM) before its implementation. The tool captured various study characteristics, including authors; years of study and publication; pathogens under investigation; study setting (country, place or region); study population; study design; pathogen identification methods (diagnostic methods); outcomes (prevalence, incidence, morbidity); pathogen strains/serotypes and AMR.

The review team was divided into three pairs, which independently extracted data from their assigned studies. Any discrepancies were resolved internally within each pair, and a third reviewer was involved to validate the extracted data.

### Risk of bias (quality) assessment

The Newcastle-Ottawa Scale tool (online supplemental appendix 2) was used to assess risk of bias (quality) in the studies.^15^ The tool evaluates three parameters (selection, comparability and outcome or exposure) in observational studies. A risk of bias score was then calculated based on specific domains for either cohort or case-control studies. A score of ≥7 indicates a low risk of bias, a score between 5 and 6 represents a medium risk of bias, and a score of ≤4 represents a high risk of bias. Each pair of reviewers on the review team (authors CMW, MY, JK, KK, CW and EM) evaluated a set of assigned studies and reconciled any discrepancies between them. In cases where conflicts arose, further consultations were conducted with a third reviewer.

### Data synthesis analysis

#### Narrative synthesis

We conducted a narrative synthesis of the studies, categorising them based on context and setting to describe the regions within each country where they were conducted. Additionally, we characterised the study population according to the setting from which participants were selected. Furthermore, we provided detailed narratives describing the methodologies employed in identifying pathogens, pathogen strains and AMR.

#### Meta-analysis and assessment of heterogeneity

Our meta-analyses were based on a random-effects model^16^ for the prevalence of each pathogen among diarrhoea cases, with a 95% CI and a significance level of 0.05. Proportions were calculated by dividing the number of events by the sample size in each study. To visually represent the pooled prevalence, forest plots were generated, while funnel plots, using the Freeman-Turkey transformation, were employed to graphically examine the distribution of studies around the pooled effects and to assess publication bias. Subgroup analyses were performed by country to facilitate comparison of the pooled prevalence within the study settings. To assess heterogeneity and identify potential outliers, Galbraith plot analysis was conducted, and heterogeneity was measured using χ^2^ heterogeneity p values and *I^2^*. Studies with an *I*^2^ of 25%–49% were interpreted as having low heterogeneity, 50%–75% moderate and 75%+ high.^17^ However, high *I*^2^ values may not solely reflect observed variation but could also be influenced by factors such as the number of studies and pooled estimates. Originally, publication bias was presumed to affect comparative studies, where those with positive results were believed to be published more frequently than those with negative results. However, this assumption does not hold true for prevalence studies without a comparison.^18 19^ Considering this, we relied solely on a funnel plot to visualise the distribution of studies around the pooled effect size. Additionally, a leave-one-out sensitivity analysis was performed to evaluate the influence of each individual study on the overall effect size. We used STATA V.18 software to perform all statistical analyses.

## Results

**Figure 1:**
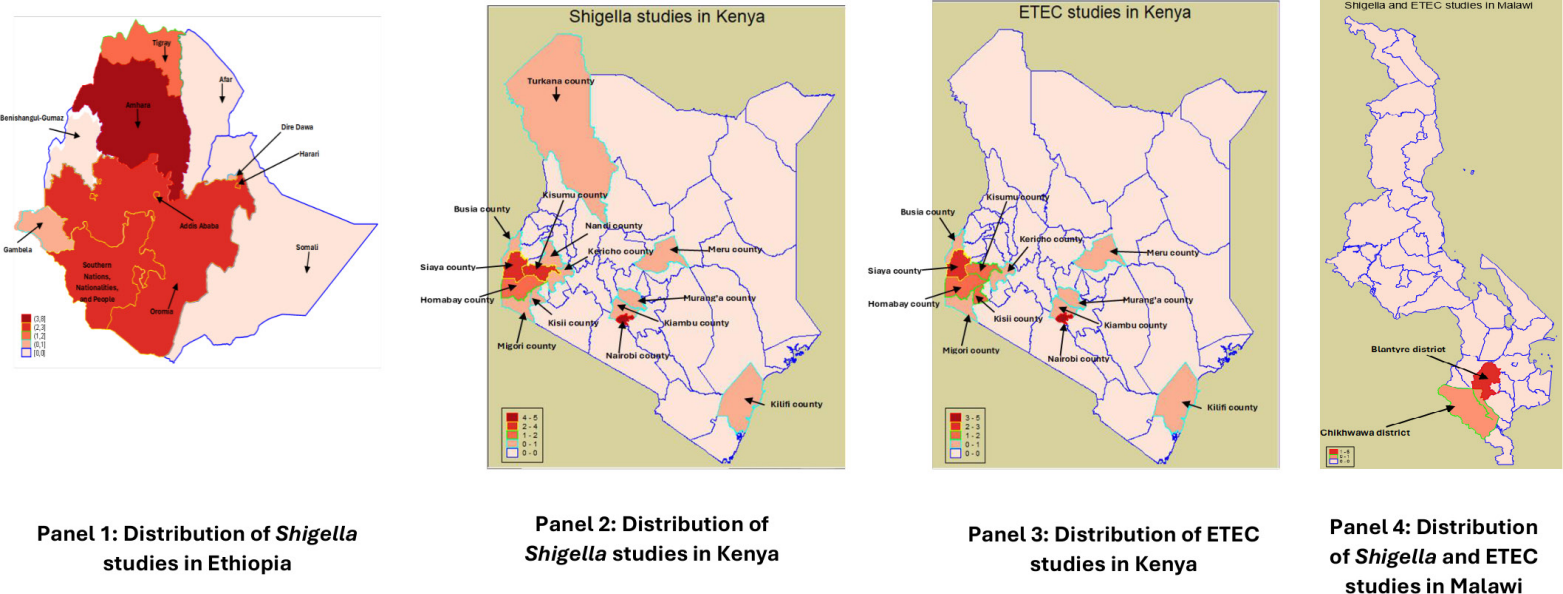
Maps showing distribution of the studies by area in each country. ETEC, enterotoxigenic *Escherichia coli*.

The review identified 3337 records across five online databases. After removing 1862 duplicate records using the Rayyan platform, 1475 records underwent initial screening based on their titles and abstracts. From these, 370 records were retrieved and reviewed for eligibility, resulting in the exclusion of 335 records. Ultimately, 35 records from the online databases were included in the final review. Additionally, 12 records were found through the references of the online records included. Among these, four records were excluded, while eight were included in the review. Consequently, a total of 43 studies were included in the final review (online supplemental appendix 4 figure 1).

### Study characteristics

Among the 43 included studies, 20 (47%) were conducted in Ethiopia, 19 (44%) in Kenya and 4 (9%) in Malawi (table 1 provides a short summary of the studies, while online supplemental appendix 3, table 1 provides more study details). Twelve (28%) studies assessed multiple pathogens, including both *Shigella* and ETEC, 21 (49%) focused on *Shigella* with other pathogens, while one (2%) was exclusively on *Shigella* and *E. coli* (with ETEC as a substrain of *E. coli*). Six (14%) studies exclusively examined *Shigella*, whereas one (2%) addressed ETEC solely. Additionally, two (5%) explored *E. coli*, with ETEC as a substrain. Therefore, a total of 40 studies focused on *Shigella*, while 16 studies included ETEC. Notably, at the time of our search, there were no ETEC studies from Ethiopia, while Kenya accounted for 81% (13/16) of the ETEC studies, and Malawi contributed three studies.

**Table 1:** Description of *Shigella* and ETEC papers

Study	Study design	Study pathogen(s)	*Shigella* pathogen identification method(s)	ETEC identification method(s)	Sample size*	*Shigella* prevalence N (%)	ETEC prevalence N (%)
**Ethiopia**							
Abebe *et al*^43^	Cross-sectional	*Shigella* and *Salmonella*	Culture	NA	204	17 (8.3%)	NA
Debas *et al*^86^	Cross-sectional	*Shigella*	Culture	NA	50	11 (22%)	NA
Dessale *et al*^25^	Cross-sectional	*Shigella* and *Salmonella*	Culture	NA	222	19 (8.6%)	NA
Balew *et al*^87^	Cross-sectional	Multiple pathogens	Culture	NA	196	8 (4%)	NA
Kahsay *et al*^88^	Cross-sectional	*Shigella*	Culture	NA	241	32 (13.3%)	NA
Getamesay *et al*^26^	Cross-sectional	*Shigella* and *Salmonella*	Culture	NA	158	11 (7%)	NA
Abera *et al*^20^	Cross-sectional	Multiple pathogens	Culture	NA	344	4 (1.2%)	NA
Ameya *et al*^44^	Cross-sectional	*Shigella* and *Salmonella*	Culture	NA	167	8 (5%)	NA
Feleke *et al*^45^	Cross-sectional	Multiple pathogens	Culture	NA	112	3 (3%)	NA
Zelelie *et al*^46^	Cross-sectional	Multiple pathogens	Culture	NA	163	3 (2%)	NA
Mekonnen *et al*^47^	Case-control	Multiple pathogens	Culture	NA	134	14 (10%)	NA
Gebreegziabher *et al*^27^	Cross-sectional	*Shigella* and *Salmonella*	Culture	NA	115	18 (16%)	NA
Mulu *et al*^89^	Surveillance	Multiple pathogens	Culture	NA	50	2 (4%)	NA
Tosisa *et al*^28^	Cross-sectional	*Shigella* and *Salmonella*	Culture	NA	239	6 (3%)	NA
Mekonnen *et al*^48^	Observational	Multiple pathogens	Culture	NA	196	11 (6%)	NA
Beyene *et al*^49^	Cross-sectional	*Shigella* and *Salmonella*	Culture	NA	260	6 (2%)	NA
Mamuye *et al*^50^	Cross-sectional	*Shigella* and *Salmonella*	Culture	NA	253	23 (9%)	NA
Ayele *et al*^51^	Cross-sectional	*Shigella*	Culture	NA	534	47 (9%)	NA
Assefa *et al*^52^	Cross-sectional	*Shigella* and *Salmonella*	Culture	NA	422	18 (4%)	NA
Admassu *et al*^29^	Cross-sectional	*Shigella* and *Salmonella*	Culture	NA	422	40 (9%)	NA
**Kenya**							
Leting *et al*^30^	Cross-sectional	*Shigella* and *Salmonella*	Culture	NA	196	18 (9%)	NA
Pavlinac *et al*^31^	Surveillance	Multiple pathogens	Culture	PCR	1360	63 (4.6%)	41 (3%)
Zachariah *et al*^53^	Cross-sectional	*Shigella* and *Campylobacter jejuni*	Culture	NA	139	28 (20%)	NA
Sang et al^23^	Cross-sectional	Multiple pathogens	Culture	PCR	651	15 (2.3%)	8 (1.2%)
Kasumba *et al*^32^	Case-control	*Shigella*	Culture	NA	1554	130 (8.4%)	NA
Beatty *et al*^33^	Surveillance	Multiple pathogens	Culture		2550	116 (5%)	
Swierczewski et al^34^	Case-control	Multiple pathogens	Culture	PCR	432	41 (9.5%)	14 (3.2%)
Njuguna *et al*^90^	Surveillance	*Shigella*	Culture		2476	389 (16%)	
Karambu *et al*^35^	Cross-sectional	Multiple pathogens	Culture	PCR	308	9 (3%)	30 (10%)
Nyanga et al^36^	Cross-sectional	*Shigella* and *E. coli*	Culture	PCR	354	14 (4%)	37 (10.5%)
Mbuthia et al^37^	Cross-sectional	Multiple pathogens	Culture	PCR	163	14 (9%)	8 (5%)
Schilling *et al*^40^	Surveillance	Multiple pathogens	Culture	PCR	1020	78 (8%)	148 (15%)
O’Reilly *et al*^91^	Surveillance	Multiple pathogens	Culture		1137	42 (4%)	
Shah *et al*^21^	Surveillance	Multiple pathogens	Culture	PCR	1060	14 (1%)	113 (11%)
Boru *et al*^38^	Case-control	Multiple pathogens	Culture	PCR	41	2 (5%)	3 (7%)
Webale *et al*^39^	Cross-sectional	Multiple pathogens	Culture	PCR	374	12 (3%)	38 (10%)
Makobe *et al*^92^	Surveillance	*E. coli*	NA	PCR	207	NA	15 (7%)
Bii *et al*^41^	Cross-sectional	*E. coli*	NA	PCR	82	NA	22 (27%)
Kipkirui *et al*^42^	Surveillance	ETEC	NA	PCR	225	NA	23 (10.2%)
**Malawi**							
Ndungo *et al*^93^	Surveillance	*Shigella*	PCR	NA	369	37 (10%)	NA
Iturriza-Gómara *et al*^*24*^	Case-control	Multiple pathogens	PCR	PCR	684	108 (16%)	213 (31%)
Versloot *et al*^22^	Cross-sectional	Multiple pathogens	PCR	PCR	47	19 (40%)	10 (21%)
Attia *et al*^94^	Cross-sectional	Multiple pathogens	PCR	PCR	64	23 (35%)	10 (16%)

* Note: The sample size refers to persons or number individuals tested

ETEC, enterotoxigenic *Escherichia coli*; NA, not applicable.

The studies were conducted across various settings, with 22 (51%) conducted in urban health facilities, 8 (19%) in rural health facilities and 10 (23%) combined both rural and urban facilities, while 3 (7%) were conducted in rural communities.

Furthermore, these studies covered different regions within each country (figure 1). In Ethiopia, most studies were conducted in the Amhara region, followed by Oromia and Southern Nations, Nationalities and Peoples. In Kenya, studies of both *Shigella* and ETEC were predominantly conducted in Nairobi County, followed by Siaya County. Meanwhile, in Malawi, most *Shigella* studies and all ETEC studies were conducted in Blantyre district, with the remaining *Shigella* study conducted in Chikhwawa district. In Malawi, all studies were conducted in the southern part of the country, with no representation from other regions. In contrast, studies in Kenya focused on the southern, western and central regions, showing a slightly broader geographic spread. Ethiopia had a more balanced distribution of studies across regions; however, none were conducted in the eastern part of the country.

All the studies included both male and female children aged under 5 years. Among the studies, 32 (74%) focused on outpatient children with diarrhoea and eight (19%) targeted hospitalised children, while three (7%) involved diarrhoeal children in the community. The study designs comprised 27 (63%) cross-sectional, 10 (23%) surveillance, five (12%) case-control and one (2%) observational study. These studies spanned various study periods ranging from 1 month (cross-sectional) to 8 years (surveillance). Likewise, the sample sizes varied, ranging from 41 to 2476 study participants.

We also examined the diagnostic techniques employed for pathogen identification. Among the 40 studies focused on *Shigella*, 36 (90%) used traditional culture methods, and 4 (10%) (all from Malawi) employed PCR methods. In contrast, ETEC identification exclusively relied on PCR methods.

### Risk of bias assessment

A detailed assessment across the reviewed studies showed a low risk of bias in 63% (27/43) of records. Conversely, 33% (14/43) of studies were categorised with a medium risk of bias. Two studies were considered to have a high risk of bias, as they lacked justifications for their sample sizes and did not adequately explain how confounders were addressed, either during study design or analysis (online supplemental appendix 3, table 2).

## Disease burden

### Shigella prevalence

**Figure 2:**
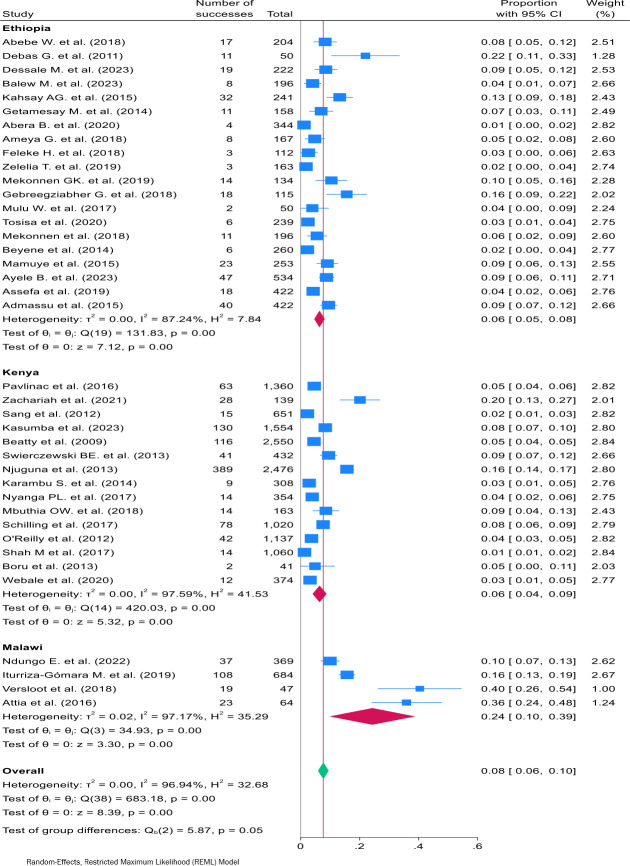
Forest plot of *Shigella* infection subgroup pooled prevalence by country, and the overall pooled prevalence among under-5 children in Ethiopia, Kenya and Malawi.

**Figure 3:**
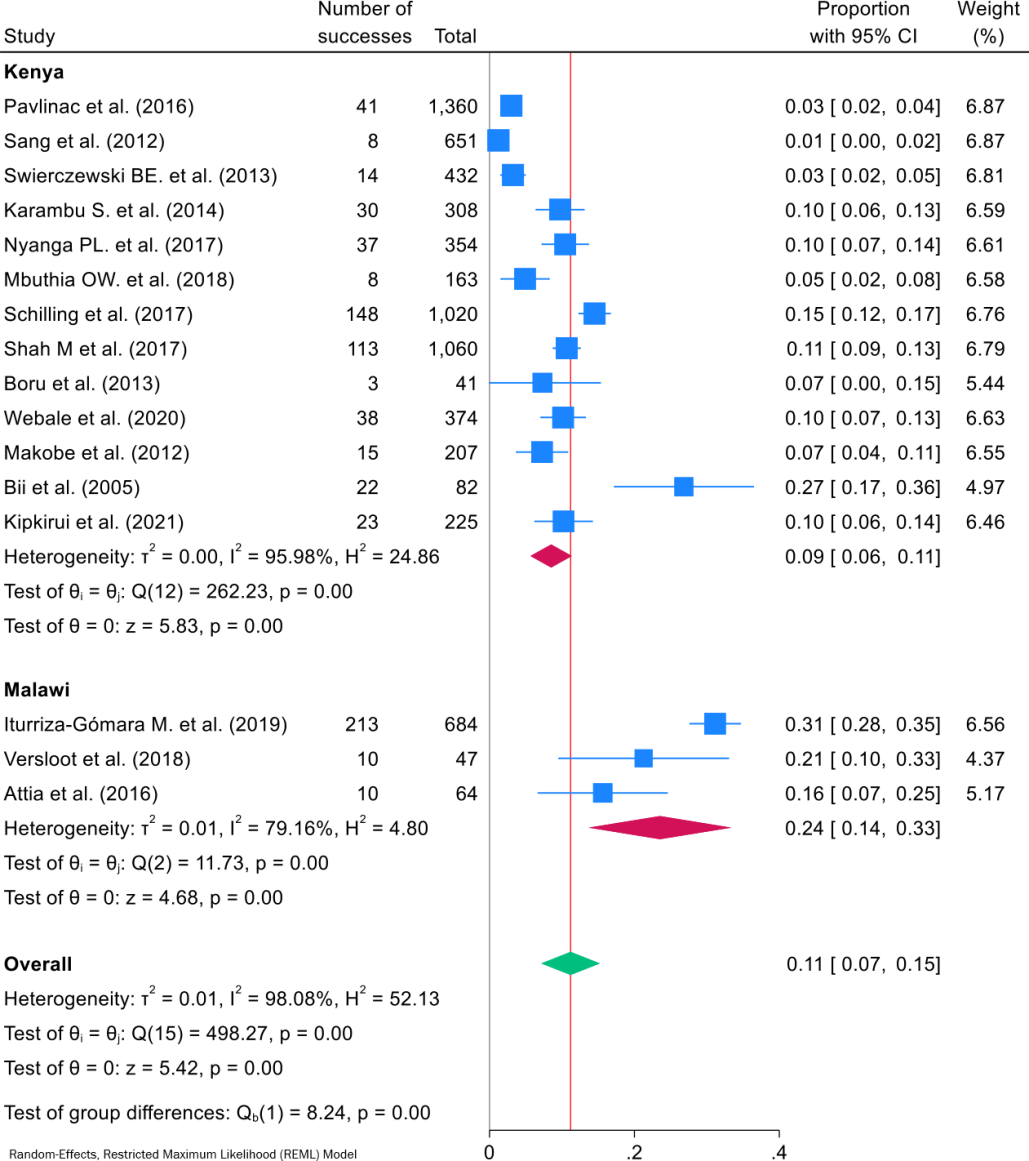
Forest plot of ETEC infection subgroup pooled prevalence by country, and the overall pooled prevalence among under-5 children in Kenya and Malawi. ETEC, enterotoxigenic *Escherichia coli*.

The prevalence of *Shigella* varied across studies, ranging from 1% (95% CI 0% to 2%^20 21^) to 40% (95% CI 26% to 54%).^22^ The subgroup analysis by country shows Malawi exhibiting a high pooled *Shigella* estimated prevalence of 24% (95% CI 10% to 39%), while in Ethiopia it was 6% (95% CI 5% to 8%) and in Kenya it was also 6% (95% CI 4% to 9%). Pooling data from the three countries, the overall prevalence of *Shigella* infection was 8% (95% CI 6% to 10%) (figure 2). Due to substantial heterogeneity (*I^2^*=96.94%, Q=683.18, p<0.001), a random-effects meta-analysis model was employed.^16^ The Galbraith plot (online supplemental appendix 4, figure 2), which illustrates standardised effect size against precision, demonstrated numerous studies falling outside the 95% confidence limits (±2), indicating substantial heterogeneity consistent with the I^2^ value. The Freeman-Turkey transformed funnel plot (online supplemental appendix 4, figure 3) displayed numerous points from individual studies scattered outside the funnel’s boundaries, particularly for studies with proportions exceeding the pooled estimate. However, the sensitivity analysis, conducted by leaving out one study at a time, indicated that each individual study did not influence the combined estimate, as all individual study estimates fell within the CI of the combined estimate (online supplemental appendix 4, figure 4).

On conducting subgroup analysis by methods of pathogen identification, PCR method of *Shigella* pathogen identification exhibited a high pooled estimated prevalence of 24% (95% CI 10% to 39%), while the pooled *Shigella* prevalence by culture method was 6% (95% CI 5% to 8%) (online supplemental appendix 4, figure 5). Notably, all studies from Malawi used PCR methods for identifying *Shigella*, while studies from Ethiopia and Kenya used culture methods. In addition, heterogeneity remained high among studies conducted within each country.

### ETEC prevalence

The prevalence of ETEC infection varied among the studies, ranging from 1% (95% CI 0% to 2%^23^) to 31% (95% CI 28% to 35%).^24^ The subgroup analysis by country showed that Malawi had a higher reported ETEC prevalence of 24% (95% CI 14% to 33%), whereas Kenya had a lower prevalence of 9% (95% CI 6% to 11%). The overall ETEC pooled prevalence was estimated to be 11% (95% CI 7% to 15%) (figure 3). Given the high degree of heterogeneity observed (I^2^=98.08%, Q=498.27, p<0.001), a random-effects meta-analysis model was used. Notably, the Galbraith plot (online supplemental appendix 4, figure 6) revealed extensive heterogeneity, with a significant number of studies falling beyond the 95% confidence limits (±2) of the plot. This finding aligns with the high I^2^ value, underscoring the substantial variability observed in the prevalence estimates across studies. The Freeman-Turkey-transformed funnel plot further displayed a number of studies distributed beyond the boundaries of the funnel (online supplemental appendix 4, figure 7). However, the sensitivity analysis indicated that each individual study had no impact on the combined estimate (online supplemental appendix 4, figure 8).

### Serotypes or species

We analysed data on pathogen strains, species or serotypes extracted from included studies that conducted pathogen isolation. Fifteen *Shigella* studies conducted bacterial isolation, with five studies from Ethiopia,^25^,^29^ 10 studies from Kenya^30^,^39^ and none from Malawi. A total of 511 *Shigella* isolates were subjected to serotyping; among these isolates, 248 (49%) were identified as *Shigella flexneri*, 116 (23%) as *S. sonnei*, 49 (10%) as *S. boydii* and 47 (9%) as *S. dysenteriae* (online supplemental appendix 3, table 3).

Among the 16 studies on ETEC, only eight provided data on the specific ETEC serotypes. Seven of these studies were conducted in Kenya,^2123 36 37 40^,^42^ whereas one study was from Malawi.^24^ Among the 562 ETEC isolates tested, 326 (58%) contained heat-stable toxins (ST), 180 (32%) contained heat-labile toxins (LT) and 56 (10%) harboured both ST and LT strains (online supplemental appendix 3, table 4).

### Antimicrobial resistance

Out of the 40 studies that reported on *Shigella*, 25 (63%) conducted antimicrobial susceptibility tests on the antibiotics commonly used for treating *Shigella* infection. Sixteen studies^2025^,^29 43^ were conducted in Ethiopia, nine^2330^,^34 36 39 53^ in Kenya and none in Malawi. Table 2 details the antimicrobials tested in both Ethiopia and Kenya, while online supplemental appendix 3, table 4 presents AMR findings from all the studies. These are descriptive rates calculated as resistant isolates over total isolates tested for each antimicrobial. Among the antimicrobials assessed in both Ethiopia and Kenya, high levels of AMR to trimethoprim-sulphamethoxazole (82.3%), doxycycline (78.6%), amoxicillin (71.1%), ampicillin (70.7%) and erythromycin (68.3%) were observed. Notably, antibiotics assessed in Kenya showed high AMR rates, including sulfisoxazole (95.7%), streptomycin (90.1%), minocycline (67.9%) and cefuroxime (53.6%), while augmentin exhibited 91% resistance and was exclusively assessed in Ethiopia (online supplemental appendix 3, table 5). Getamasay *et al*^26^ and Assefa *et al*^52^ reported a 100% multidrug resistance rate among the isolates, while Ayele *et al*^51^ and Dessale *et al*^25^ documented rates of 85% and 84%, respectively. Pavlinic *et al*^31^ reported a lower MDR rate of 35%.

**Table 2:** *Shigella* antimicrobial resistance by country

Classification	Antibiotics	Ethiopia		Kenya		Overall	
		Isolates tested	Resistant isolates N (%)	Isolates tested	Resistant isolates N (%)	Isolates tested	Resistant isolates N (%)
Penicillins	Amoxicillin	194	167 (86)	169	91 (53.8)	363	258 (71.1)
	Ampicillin	222	197 (88.7)	480	299 (62.3)	702	496 (70.7)
	Augmentin	23	21 (91)	–	–	23	21 (91)
Cephalosporins	Ceftriaxone	129	12 (9.3)	357	22 (6.2)	486	34 (7)
	Cefuroxime	–	–	28	15 (53.6)	28	15 (53.6)
	Cephalexin	4	1 (25)	–	–	4	1 (25)
	Cefotaxime	6	0 (0)	–	–	6	0 (0)
	Ceftizoxime	40	11 (27.5)	–	–	40	11 (27.5)
	Cefoxitin	47	10 (21.3)	–	–	47	10 (21.3)
	Cephalotin	14	0 (0)	–	–	14	0 (0)
	Ceftazidime	35	3 (8.6)	18	3 (16.7)	88	6 (6.8)
Quinolones and fluoroquinolones	Norfloxacin	130	6 (4.6)	43	10 (23.3)	173	16 (9.2)
	Ciprofloxacin	221	19 (8.6)	302	17 (5.6)	523	36 (6.9)
	Nalidixic acid	142	21 (14.8)	354	23 (6.5)	496	44 (8.9)
Aminoglycosides	Kanamycin	33	0 (0)	151	0 (0)	184	0 (0)
	Amikacin	6	0 (0)	18	1 (5.6)	24	1 (4.2)
	Streptomycin	–	–	151	136 (90.1)	151	136 (90.1)
	Gentamicin	189	47 (24.9)	212	12 (5.7)	401	59 (14.7)
Macrolides	Erythromycin	82	71 (86.6)	63	28 (44.4)	145	99 (68.3)
	Azithromycin	19	8 (42.1)	160	1 (0.6)	179	9 (5)
Tetracyclines	Tetracycline	188	134 (71.3)	274	223 (81.4)	462	357 (77.3)
	Doxycycline	84	65 (77.4)	28	23 (82.1)	112	88 (78.6)
	Minocycline	–	–	28	19 (67.9)	20	19 (67.9)
Chloramphenicol	Chloramphenicol	248	115 (46.4)	166	91 (54.8)	414	206 (49.8)
Lincosamides	Clindamycin	8	3 (37.5)	–	–	8	3 (37.5)
Sulphonamides	Sulfisoxazole	–	–	116	111 (95.7)	116	111 (95.7)
	Trimethoprim-sulphamethoxazole	234	119 (50.9)	480	414 (86.3)	648	533 (82.3)

However, in both Ethiopia and Kenya, all *Shigella* isolates demonstrated 100% susceptibility to kanamycin and high susceptibility rates to other antimicrobials, including amikacin (95.8%), azithromycin (95%), ceftazidime (93.2%), ciprofloxacin (93.1%), nalidixic acid (91.1%) and norfloxacin (90.8%). In Ethiopia, additional antimicrobials were assessed, revealing that *Shigella* isolates displayed 100% susceptibility to cephalothin and cefotaxime, while cephalexin and gentamicin exhibited 75% and 78.7% susceptibility, respectively.

Only one study conducted in Kenya^34^ provided data on ETEC resistance for children under the age of 5 years. Among the 20 isolates examined, 18 (90%) displayed resistance to ampicillin, 15 (75%) to trimethoprim-sulfamethoxazole, 14 (70%) to tetracycline and 5 (20%) to ciprofloxacin.

## Discussion

Diarrhoeal diseases pose a significant challenge in LMICs, particularly among children under the age of 5 years, resulting in high morbidity and mortality, including long-term effects of malnutrition such as stunting.^54^ While various pathogens contribute to diarrhoea, this review specifically addresses the burden caused by *Shigella* and ETEC in Ethiopia, Kenya and Malawi, amidst growing concerns of AMR.

This review found an 8% pooled prevalence of *Shigella* among children under 5 with diarrhoea, with country-level estimates ranging from 6% in Ethiopia and Kenya to 24% in Malawi. The higher prevalence in Malawi may reflect the inclusion, in two studies of children with severe acute malnutrition, a population at increased risk of infection. However, the wide CI for the Malawi estimate, driven by limited studies, indicates substantial uncertainty. Notably, our overall pooled estimate aligns with the Vaccine Impact on Diarrhoea in Africa study’s reported 7% prevalence of *Shigella* in the Gambia, Mali and Kenya,^32^ supporting regional consistency despite methodological differences. A systematic review by Ayele *et al*^55^ encompassing all age groups in East Africa also determined a similar prevalence of *Shigella* at 6%, reaffirming the high prevalence of *Shigella* among younger children in low-resource settings as noted by Rogawaski *et al*.^56^ A recent systematic review by Nyarkoh *et al*^57^ reported an all-age *Shigella* prevalence of 6% in Africa, with 6.2% in East Africa (including Ethiopia, Kenya and Malawi) and 6% among children under 5. Our pooled estimate of 8% suggests an increase from the 4% previously reported by Lanata *et al*,^58^ likely reflecting shifts in enteric pathogen epidemiology following rotavirus vaccine introduction, which reduced rotavirus-associated diarrhoea cases and may have heightened the relative burden of other pathogens like *Shigella*.^59^

The Global Burden of Disease (GBD) 2021 data indicate that *Shigella* contributes disproportionately to diarrhoea-related years lived with disability (YLDs) among children under 5 in East Africa (20% overall), with a higher burden in Ethiopia (26%) and Kenya (16%)^60 61^ than expected based on prevalence alone. This discrepancy may stem from the limited sensitivity of culture-based diagnostics (the primary method in included studies), variations in pathogen virulence, coinfections or healthcare access.

For ETEC, the pooled prevalence in this study was 11%, ranging from 7% in Kenya to 24% in Malawi. Our pooled estimate exceeds the 5% prevalence previously reported for Africa by Lanata *et al.*^58^ However, this estimate of 11% aligns closely with the GBD-estimated 13% of diarrhoea-related YLDs attributed to ETEC. However, country-level variations exist (11% in Kenya vs 10% in Malawi),^60 61^ possibly reflecting differences in disease severity or study populations. However, the differences between our empirical data and GBD’s modelled estimates may reflect regional heterogeneity in surveillance or diagnostic methods, warranting further investigation.

This study is consistent in demonstrating the superior sensitivity of PCR techniques over traditional culture-dependent methods in *Shigella* pathogen identification. PCR techniques can detect and differentiate pathogen serotypes and AMR genes, offering a comprehensive approach for disease surveillance.^62 63^ However, assessing morbidity or mortality attributable to specific diarrhoeal pathogens is complicated by limited infrastructure in LMICs.^64^ Implementing sensitive PCR requires significant investment in funding, laboratory infrastructure and skilled personnel, presenting difficulties for resource-limited countries. Consequently, these countries often rely on less-sensitive culture-dependent methods,^64^ implying that the burdens of *Shigella* or ETEC diarrhoea in LMICs are likely underestimated or undetected, potentially reducing attention in public health agendas.

The notably higher *Shigella* prevalence in Malawi can be attributed to the use of PCR techniques in the reviewed studies, whereas culture-based methods were employed in Ethiopia and Kenya. Lindsay *et al*^65^ corroborated this observation, noting that estimates of *Shigella* prevalence are affected by the inconsistent sensitivity of diagnostic and surveillance methods, alongside contextual factors such as geographical and environmental conditions. Their study demonstrated a 90% increase in *Shigella* detection with PCR relative to 10% with culture techniques in the same samples.

While ETEC is recognised as a significant contributor to the global burden of diarrhoeal diseases, especially in LMICs, recent epidemiological studies have highlighted substantial regional variations in morbidity and mortality estimates attributable to this pathogen.^4 66^ In Africa, these estimates have predominantly relied on culture-based diagnostic methods, which are limited in their ability to detect ETEC due to their inability to distinguish pathogenic strains from commensal *E. coli* in the absence of molecular analysis.^67^ For instance, *Liu et al*^68^ demonstrated the superiority of molecular diagnostics by reanalysing samples from the Global Enteric Multicenter Study. Using quantitative real-time PCR (qPCR), they showed that the attributable incidence of most pathogens, including ETEC, was significantly higher than previously estimated using conventional culture techniques. Specifically, ETEC detection increased by approximately 1.5-fold, as culture methods failed to identify strains producing the heat-stable toxin subtype STp, thereby underestimating the true burden of ST-ETEC. As a result, qPCR positioned ETEC among the pathogens with the highest attributable fractions for childhood diarrhoea.

However, our review reveals the challenges of employing PCR-based diagnostics in LMIC settings, particularly in the limited data availability. Out of the 18 studies analysed, 15 were conducted in Kenya, three in Malawi and none from Ethiopia. Among these studies, only two exclusively focused on ETEC, while the rest assessed multiple enteric pathogens, identifying ETEC only as a subtype of *E. coli*. The absence of ETEC studies in Ethiopia and the limited ETEC-specific studies in our review highlights significant diagnostic and surveillance gaps in LMICs. Thus, LMIC governments should invest in diagnostic infrastructure, strengthen surveillance systems and support national research institutions in prioritising ETEC within their research agendas. Kenya serves as a noteworthy example of how sustained investments and strategic mobilisation of resources can establish a robust research ecosystem in similar settings.^69^

Capturing pathogen-prevalence estimates informs the estimation of other factors, such as AMR and pathogen subtypes. This review has established AMR among *Shigella* isolates to various antibiotic classes commonly used to treat *Shigella* infections in children under the age of 5 years. Specifically, high levels of AMR were observed in penicillins, tetracyclines, macrolides and sulphonamides. A comparison with findings from Ayele *et al*,^55^ which included adult populations, reveals similar resistance patterns, with *Shigella* isolates demonstrating resistance to tetracycline, ampicillin, amoxicillin, chloramphenicol and trimethoprim-sulfamethoxazole. However, both studies found susceptibility to quinolones and fluoroquinolones (such as norfloxacin, nalidixic acid and ciprofloxacin) and cephalosporins (such as ceftriaxone). Kalule *et al*,^69^ however, in their recent systematic review, indicated slightly higher AMR among *Shigella* isolates to quinolones and cephalosporins, raising concerns about the potential emergence of high resistance in these antibiotic classes due to overuse and misuse, particularly in LMICs.^70 71^ Ciprofloxacin is recommended by the WHO as the first-line treatment for Shigella dysentery, with azithromycin, cefixime, ceftriaxone and trimethoprim-sulfamethoxazole identified as second-line options.^6^ Our review revealed varied use of these antibiotics across the two countries studied, with higher levels of AMR observed for second-line drugs such as trimethoprim-sulfamethoxazole compared with ciprofloxacin, the first-line option. Meanwhile, only one study in this review reported AMR among ETEC isolates, which exhibited similar resistance patterns observed for *Shigella*, with high resistance to ampicillin, trimethoprim-sulfamethoxazole and tetracycline, and susceptibility to ciprofloxacin. Although the review did not directly capture antibiotic prescription patterns, the range of antibiotics identified suggests empirical prescribing. Such practices are likely to contribute to the growing AMR burden. These findings highlight the urgent need for complementary strategies, such as vaccines, to reduce reliance on antibiotics and curb AMR while mitigating the burden of diarrhoeal diseases in high-risk settings.

Our analysis shows that among the ETEC isolates, those producing ST enterotoxins were the most prevalent, followed by those producing LT enterotoxins. While for *Shigella* species, *S. flexneri* emerged as the primary cause of shigellosis in the three countries, followed by *S. sonnei, S. boydii* and *S. dysenteriae*. This pattern is consistent with earlier findings by Kasumba *et al*^32^ and Ayele *et al*,^55^ who also identified *S. flexneri* as a commonly isolated *Shigella* serogroup in SSA. *S. flexneri* is recognised as an endemic species that is particularly prevalent in resource-constrained settings, with its incidence typically declining as economic development improves, while the relative prevalence of *S. sonnei* tends to increase.^72^ Ayele *et al*^55^ further reported that *S. flexneri* was the most resistant *Shigella* serogroup, followed by *S. dysenteriae.* The similarity of ETEC and *Shigella* AMR patterns is particularly concerning, as it reduces the range of effective treatment options available for these infections.

A strategic approach for tackling the burden of diarrhoeal diseases involves the generation of inclusive evidence. Contextual evidence, providing a localised perspective, is crucial for policymakers in making informed decisions.^73^ It is essential to comprehensively gather such evidence, encompassing a wide geographical range, from regional to national levels.^74^ Our analysis reveals a concentration of research studies in specific areas and regions within countries, particularly evident in Malawi. It is recommended that future research encompasses diverse regions within countries. This approach facilitates a nuanced understanding of local contexts, facilitates tailored interventions, promotes equity and fosters engagement with local stakeholders, all crucial for effective policy implementation. Collaborative efforts and efficient resource utilisation are pivotal in achieving this goal.^75^ Furthermore, LMICs stand to benefit significantly from bolstering their National Public Health Institutes (NPHIs) through their Ministries of Health. Strengthening NPHIs enables them to enhance disease surveillance, laboratory capabilities and health data analysis, thus providing vital inputs for shaping national health policies.^76^

Our review highlights the disease burdens of *Shigella* and ETEC-related diarrhoea, prompting the need for complementary interventions beyond improved hygiene and sanitation, most notably the development of effective vaccines. The successful introduction of the rotavirus vaccine into national Expanded Programmes on Immunisation, following the 2009 WHO recommendation, has significantly reduced rotavirus-associated diarrhoea hospitalisations in countries that adopted the vaccine, including Ethiopia, Kenya and Malawi.^77^,^80^ This success illustrates the potential impact of additional vaccines targeting *Shigella* and ETEC in similarly high-burden settings. Currently, no licensed vaccines exist for *Shigella* and ETEC. Nevertheless, several promising vaccine candidates have advanced through various stages of preclinical and clinical development.^81^ Progress in vaccine development hinges greatly on heightening awareness of the disease burden posed by *Shigella* and ETEC, which is essential for securing augmented funding.^82^ Furthermore, comprehending the diversity of *Shigella* and ETEC subtypes is pivotal for designing vaccines that strike a balance between subtype, species or strain coverage, safety and immunogenicity, thereby effectively combating these pathogens.^83 84^ Moreover, there is a pressing demand for comprehensive data on the disease burden attributable to *Shigella* and ETEC pathogens, along with effective communication of this information to relevant stakeholders, including policymakers at the country level. This understanding is vital for motivating the introduction of vaccines against these pathogens once they become available, as noted by Fleming *et al*.^85^ Our review contributes to addressing this crucial need for information.

### Limitations

We encountered challenges during the data extraction process for eligible robust multicounty studies. These studies aggregated data in their analysis and presentation, making it difficult to isolate country-specific data. Additionally, since our study focused on children under the age of 5 years, some studies did not conduct age-specific analyses, particularly if they included older age groups. Similarly, in some multipathogen studies that assessed AMR, the results were not presented for pathogen-specific AMR.

Our review was limited to published studies, and it would have been valuable to include grey literature or surveillance reports from each country to supplement our findings. However, our analysis revealed that routine surveillance for *Shigella* and ETEC may not be fully established and/or relied mostly on culture methods that may underestimate the burden of pathogen-specific diarrhoeal diseases. Therefore, assessing national disease surveillance systems would be beneficial to understand the indicators and analytics collected for diarrhoeal epidemiology in children under the age of 5.

Another limitation is the geographical focus of our review, which is limited to Ethiopia, Kenya and Malawi. This scope may not fully capture the broader picture of *Shigella* and ETEC across other regions, particularly in SSA. Therefore, expanding future reviews to include the wider SSA region could offer a more holistic view of *Shigella* and ETEC and their impact.

## Conclusion

Our review highlights the significant burden of *Shigella* and ETEC pathogens on children under the age of 5 years in Malawi, Kenya and Ethiopia. The review suggests that the burden of diarrhoea attributed to *Shigella* may be higher than reported due to limitations in pathogen identification methods, particularly due to the reliance on culture-based techniques. Therefore, using standardised and sensitive PCR techniques in studies and disease surveillance in LMICs can enhance understanding and monitoring of these two pathogens, aiding in the development of effective interventions.

The levels of antibiotic resistance identified in this review highlight the threat of AMR and the need for immediate stewardship and preventative strategies to address it. Vaccination presents a promising approach to directly reduce the burden of diarrhoea caused by *Shigella* and ETEC while indirectly mitigating the challenge of AMR by decreasing antibiotic use.

## Supplementary material

10.1136/bmjgh-2024-018515 online supplemental file 1

10.1136/bmjgh-2024-018515 online supplemental file 2

10.1136/bmjgh-2024-018515 online supplemental file 3

10.1136/bmjgh-2024-018515 online supplemental file 4

## Data Availability

All data relevant to the study are included in the article or uploaded as supplementary information.
