# Supplementary material for: Burden of Shigella and enterotoxigenic Escherichia coli infections among children under 5 years in Ethiopia, Kenya and Malawi: a systematic review and meta-analysis
Source: BMJ Glob Health. 2026 Mar 2;11(3):e018515. doi: 10.1136/bmjgh-2024-018515 (PMC12958989; doi:10.1136/bmjgh-2024-018515)
Supplement: online supplemental file 1 [file bmjgh-11-3-s001.docx]

**Supplementary appendix 1: Search strategy**

| **Database** | **Number of results** |
| --- | --- |
| Web of Science | 777 |
| African Index Medicus | 30 |
| Medline (Ovid) | 936 |
| CINAHL | 344 |
| PubMed | 1250 |
| TOTAL | 3,337 |

All searches completed by CM on 28^th^ July 2023

**Web of Science**

# Searches:

1: (((((((ALL=("Children under the age of five years")) OR ALL=("Under five children")) OR

ALL=(Under-five*)) OR ALL=(Infant*)) OR ALL=("Pre-school child*")) OR ALL=(Neonate*)) OR

ALL=(Newborn*)) OR ALL=(Babies) Date Run: Fri Jul 28 2023 11:19:08 GMT+0100 (British

Summer Time) Results: 855622

2: (((((((((((((ALL=(Diarrh*)) OR ALL=("Diarrh* disease*")) OR ALL=(Gastroenteritis)) OR

ALL=("Gastrointestinal infection*")) OR ALL=("Gastro-intestinal infection*")) OR

ALL=(Rotavirus)) OR ALL=("Rotavirus infection*")) OR ALL=(Shigella)) OR ALL=("Bacillary

dysentery")) OR ALL=(Dysentery)) OR ALL=(Shigellosis)) OR ALL=("Enterotoxigenic

escherichia coli")) OR ALL=("Escherichia coli")) OR ALL=(ETEC) Date Run: Fri Jul 28 2023

11:19:09 GMT+0100 (British Summer Time) Results: 683059

3: (((((((((((((ALL=("Disease burden")) OR ALL=("Burden of disease")) OR ALL=(Hospitalization))

OR ALL=(Hospitalisation)) OR ALL=(Inpatient*)) OR ALL=(Outpatient*)) OR

ALL=(Epidemiology)) OR ALL=(Prevalence)) OR ALL=(Incidence)) OR ALL=(Morbidity)) OR

ALL=(Mortality)) OR ALL=(Death)) OR ALL=(Illness)) OR ALL=(Sickness) Date Run: Fri Jul

28 2023 11:19:09 GMT+0100 (British Summer Time) Results: 5095541

4: ((ALL=(Malawi)) OR ALL=(Kenya)) OR ALL=(Ethiopia) Date Run: Fri Jul 28 2023

11:19:09 GMT+0100 (British Summer Time) Results: 187872

5: #1 AND #2 AND #3 AND #4 Timespan: 2000-01-01 to 2030-01-01 Date Run: Fri Jul 28

2023 11:19:10 GMT+0100 (British Summer Time) Results: 777

**Search strategy for the African Index Medicus**

tw:(tw:((tw:("Children under the age of five years" OR "Under five children"or under-five* OR infant* OR "Preschool child*" OR neonate* OR newborn* OR babies)) AND (tw:(diarrh* OR "Diarrh* disease*" OR gastroenteritis OR "Gastrointestinal infection*" OR "Gastro-intestinal infection*" OR rotavirus OR "Rotavirus infection*" OR shigella OR "Bacillary dysentery" OR dysentery OR shigellosis OR "Enterotoxigenic escherichia coli" OR "Escherichia coli" OR etec)) AND (tw:("Disease burden" OR "Burden of disease" OR hospitalization OR hospitalisation OR inpatient* OR outpatient* OR epidemiology OR prevalence OR incidence OR morbidity OR mortality OR death OR illness OR sickness)) AND (tw:(malawi OR kenya OR ethiopia))) AND (year_cluster:[2000 TO 2030])) AND (year_cluster:[2000 TO 2030])

**Ovid MEDLINE(R) ALL <1946 to July 27, 2023>**

1 "Children under the age of five years".mp. 244

2 Under-five*.mp. 8193

3 "Under five".mp. 7807

4 exp Infant/ or Infant*.mp. 1396491

5 "Infant, newborn".mp. or exp Infant, Newborn/ 674446

6 Neonate*.mp. 108935

7 Babies.mp. 42153

8 exp Child, Preschool/ or "Pre-school child*".mp. 990871

9 Newborn*.mp. 836700

10 1 or 2 or 3 or 4 or 5 or 6 or 7 or 8 or 9 2031990

11 exp Diarrhea/ or Diarrh*.mp. 142822

12 Gastroenteritis.mp. or exp Gastroenteritis/ 260015

13 "Gastrointestinal infection*".mp. 2958

14 "Gastro-intestinal infection*".mp. 103

15 Rotavirus.mp. or exp Rotavirus/ 17353

16 exp Rotavirus Infections/ 8710

17 Shigella.mp. or exp Shigella/ 19458

18 "Bacillary Dysentery".mp. or exp Dysentery, Bacillary/ 8436

19 Shigellosis.mp. 2508

20 "Enterotoxigenic escherichia coli".mp. or exp Enterotoxigenic Escherichia coli/ 3671

21 exp Escherichia coli Infections/ or "Escherichia coli infection*".mp. 36178

22 "Escherichia coli".mp. or exp Escherichia coli/ 426474

23 ETEC.mp. 2963

24 11 or 12 or 13 or 14 or 15 or 16 or 17 or 18 or 19 or 20 or 21 or 22 or 23 807461

25 10 and 24 75446

26 "Disease burden".mp. 22385

27 "Burden of disease*".mp. 16614

28 Hospitalization.mp. or exp Hospitalization/ 404826

29 Hospitalisation.mp. 21721

30 exp Inpatients/ or Inpatient*.mp. 150413

31 exp Outpatients/ or Outpatient*.mp. 224251

32 Epidemiology.mp. or exp Epidemiology/ 2229234

33 exp Prevalence/ or Prevalence.mp. 883971

34 Incidence.mp. or exp Incidence/ 1027175

35 Morbidity.mp. or exp Morbidity/ 1053784

36 exp Mortality/ or Mortality.mp. 1525341

37 Death.mp. or exp Death/ 1040632

38 Illness.mp. 593759

39 Sickness.mp. 41030

40 26 or 27 or 28 or 29 or 30 or 31 or 32 or 33 or 34 or 35 or 36 or 37 or 38 or 39 5749128

41 25 and 40 36192

42 Malawi.mp. or exp Malawi/ 9832

43 Kenya.mp. or exp Kenya/ 27191

44 Ethiopia.mp. or exp Ethiopia/ 30238

45 42 or 43 or 44 65551

46 41 and 45 1118

47 limit 46 to yr="2000 -Current" 936

**CINAHL**

Wednesday, July 26, 2023 11:11:49 PM

| **#** | **Query** | **Limiters/Expanders** | **Last Run Via** | **Results** |
| --- | --- | --- | --- | --- |
| S49 | S43 AND S47 | Limiters - Publication Year: 2000-2030  Expanders - Apply  equivalent subjects | Interface - EBSCOhost Research Databases Search Screen - Advanced Search | 344 |
|  |  | Search modes - Find all my search terms | Database - CINAHL Plus |  |
| S48 | S43 AND S47 | Expanders - Apply equivalent subjects | Interface - EBSCOhost Research Databases | 354 |
|  |  | Search modes - Find all my search terms | Search Screen - Advanced Search  Database - CINAHL Plus |  |
| S47 | S44 OR S45 OR S46 | Expanders - Apply | Interface - EBSCOhost | 20,502 |
|  |  | equivalent subjects Search modes - Find all my search terms | Research Databases Search Screen - Advanced Search  Database - CINAHL Plus |  |
| S46 | (MH "Ethiopia") OR "Ethiopia" | Expanders - Apply equivalent subjects Search modes - Find all my search terms | Interface - EBSCOhost Research Databases Search Screen - Advanced Search | 8,624 |
|  |  |  | Database - CINAHL Plus |  |
| S45 | (MH "Kenya") OR "Kenya" | Expanders - Apply equivalent subjects Search modes - Find all | Interface - EBSCOhost Research Databases Search Screen - Advanced | 8,567 |
|  |  | my search terms | Search  Database - CINAHL Plus |  |
| S44 | (MH "Malawi") OR "Malawi" | Expanders - Apply equivalent subjects | Interface - EBSCOhost Research Databases | 3,853 |
|  |  | Search modes - Find all my search terms | Search Screen - Advanced Search  Database - CINAHL Plus |  |
| S43 | S27 AND S42 | Expanders - Apply | Interface - EBSCOhost | 8,422 |
|  |  | equivalent subjects Search modes - Find all my search terms | Research Databases Search Screen - Advanced Search  Database - CINAHL Plus |  |

| S42 | S28 OR S29 OR S30 OR S31 OR S32 OR S33 OR | Expanders - Apply equivalent subjects | Interface - EBSCOhost Research Databases | 1,924,736 |
| --- | --- | --- | --- | --- |
|  | S34 OR S35 OR S36 OR S37 OR S38 OR S39 OR S40 OR S41 | Search modes - Find all my search terms | Search Screen - Advanced Search  Database - CINAHL Plus |  |
| S41 | "Sickness" | Expanders - Apply | Interface - EBSCOhost | 17,192 |
|  |  | equivalent subjects Search modes - Find all my search terms | Research Databases Search Screen - Advanced Search  Database - CINAHL Plus |  |
| S40 | "Illness" | Expanders - Apply equivalent subjects Search modes - Find all my search terms | Interface - EBSCOhost Research Databases Search Screen - Advanced Search | 249,644 |
|  |  |  | Database - CINAHL Plus |  |
| S39 | (MH "Death+") OR  "Death" | Expanders - Apply equivalent subjects Search modes - Find all | Interface - EBSCOhost Research Databases Search Screen - Advanced | 234,505 |
|  |  | my search terms | Search  Database - CINAHL Plus |  |
| S38 | (MH "Mortality+") OR "Mortality" | Expanders - Apply equivalent subjects | Interface - EBSCOhost Research Databases | 360,720 |
|  |  | Search modes - Find all my search terms | Search Screen - Advanced Search  Database - CINAHL Plus |  |
| S37 | (MH "Morbidity+") OR | Expanders - Apply | Interface - EBSCOhost | 290,704 |
|  | "Morbidity" | equivalent subjects Search modes - Find all my search terms | Research Databases Search Screen - Advanced Search  Database - CINAHL Plus |  |
| S36 | (MH "Incidence") OR "Incidence" | Expanders - Apply equivalent subjects Search modes - Find all my search terms | Interface - EBSCOhost Research Databases Search Screen - Advanced Search | 235,734 |
|  |  |  | Database - CINAHL Plus |  |
| S35 | (MH "Prevalence") OR "Prevalence" | Expanders - Apply equivalent subjects Search modes - Find all | Interface - EBSCOhost Research Databases Search Screen - Advanced | 265,418 |
|  |  | my search terms | Search  Database - CINAHL Plus |  |
| S34 | (MH "Epidemiology+") OR "Epidemiology" | Expanders - Apply equivalent subjects | Interface - EBSCOhost Research Databases | 1,062,810 |

|  |  | Search modes - Find all my search terms | Search Screen - Advanced Search  Database - CINAHL Plus |  |
| --- | --- | --- | --- | --- |
| S33 | (MH "Outpatients") OR "Outpatient*" | Expanders - Apply equivalent subjects Search modes - Find all | Interface - EBSCOhost Research Databases Search Screen - Advanced | 114,806 |
|  |  | my search terms | Search  Database - CINAHL Plus |  |
| S32 | (MH "Inpatients") OR "Inpatient*" | Expanders - Apply equivalent subjects | Interface - EBSCOhost Research Databases | 132,292 |
|  |  | Search modes - Find all my search terms | Search Screen - Advanced Search  Database - CINAHL Plus |  |
| S31 | "Hospitalisation" | Expanders - Apply | Interface - EBSCOhost | 25,708 |
|  |  | equivalent subjects Search modes - Find all my search terms | Research Databases Search Screen - Advanced Search  Database - CINAHL Plus |  |
| S30 | (MH "Hospitalization+") OR "Hospitalization" | Expanders - Apply equivalent subjects Search modes - Find all my search terms | Interface - EBSCOhost Research Databases Search Screen - Advanced Search | 160,429 |
|  |  |  | Database - CINAHL Plus |  |
| S29 | ""Burden of disease"" | Expanders - Apply equivalent subjects Search modes - Find all | Interface - EBSCOhost Research Databases Search Screen - Advanced | 51,362 |
|  |  | my search terms | Search  Database - CINAHL Plus |  |
| S28 | ""Disease burden"" | Expanders - Apply equivalent subjects | Interface - EBSCOhost Research Databases | 51,362 |
|  |  | Search modes - Find all my search terms | Search Screen - Advanced Search  Database - CINAHL Plus |  |
| S27 | S10 AND S26 | Expanders - Apply | Interface - EBSCOhost | 14,092 |
|  |  | equivalent subjects Search modes - Find all my search terms | Research Databases Search Screen - Advanced Search  Database - CINAHL Plus |  |
| S26 | S11 OR S12 OR S13 OR S14 OR S15 OR S16 OR S17 OR S18 OR S19 OR | Expanders - Apply equivalent subjects Search modes - Find all my search terms | Interface - EBSCOhost Research Databases Search Screen - Advanced | 79,705 |

|  | S20 OR S21 OR S22 OR S23 OR S24 OR S25 |  | Search  Database - CINAHL Plus |  |
| --- | --- | --- | --- | --- |
| S25 | "ETEC" | Expanders - Apply equivalent subjects Search modes - Find all my search terms | Interface - EBSCOhost Research Databases Search Screen - Advanced Search | 159 |
|  |  |  | Database - CINAHL Plus |  |
| S24 | (MH "Escherichia Coli") OR ""Escherichia coli"" | Expanders - Apply equivalent subjects Search modes - Find all | Interface - EBSCOhost Research Databases Search Screen - Advanced | 11,962 |
|  |  | my search terms | Search  Database - CINAHL Plus |  |
| S23 | (MH "Escherichia Coli Infections") OR | Expanders - Apply equivalent subjects | Interface - EBSCOhost Research Databases | 3,646 |
|  | ""Enterotoxigenic escherichia coli"" | Search modes - Find all my search terms | Search Screen - Advanced Search  Database - CINAHL Plus |  |
| S22 | "Shigellosis" | Expanders - Apply | Interface - EBSCOhost | 465 |
|  |  | equivalent subjects Search modes - Find all my search terms | Research Databases Search Screen - Advanced Search  Database - CINAHL Plus |  |
| S21 | (MH "Dysentery+") OR "Dysentery" | Expanders - Apply equivalent subjects Search modes - Find all my search terms | Interface - EBSCOhost Research Databases Search Screen - Advanced Search | 1,080 |
|  |  |  | Database - CINAHL Plus |  |
| S20 | (MH "Dysentery, Bacillary") OR ""Bacillary dysentery"" | Expanders - Apply equivalent subjects Search modes - Find all | Interface - EBSCOhost Research Databases Search Screen - Advanced | 475 |
|  |  | my search terms | Search  Database - CINAHL Plus |  |
| S19 | (MH "Shigella") OR "Shigella" | Expanders - Apply equivalent subjects | Interface - EBSCOhost Research Databases | 1,074 |
|  |  | Search modes - Find all my search terms | Search Screen - Advanced Search  Database - CINAHL Plus |  |
| S18 | (MH "Rotavirus | Expanders - Apply | Interface - EBSCOhost | 1,929 |
|  | Infections") OR ""Rotavirus infection*"" | equivalent subjects Search modes - Find all my search terms | Research Databases Search Screen - Advanced Search  Database - CINAHL Plus |  |

| S17 | (MH "Rotaviruses") OR "Rotavirus" | Expanders - Apply equivalent subjects | Interface - EBSCOhost Research Databases | 2,597 |
| --- | --- | --- | --- | --- |
|  |  | Search modes - Find all my search terms | Search Screen - Advanced Search  Database - CINAHL Plus |  |
| S16 | ""Gastro-intestinal | Expanders - Apply | Interface - EBSCOhost | 104 |
|  | infection*"" | equivalent subjects Search modes - Find all my search terms | Research Databases Search Screen - Advanced Search  Database - CINAHL Plus |  |
| S15 | ""Gastrointestinal infection*"" | Expanders - Apply equivalent subjects Search modes - Find all my search terms | Interface - EBSCOhost Research Databases Search Screen - Advanced Search | 8,439 |
|  |  |  | Database - CINAHL Plus |  |
| S14 | (MH "Gastroenteritis+") OR "Gastroenteritis" | Expanders - Apply equivalent subjects Search modes - Find all | Interface - EBSCOhost Research Databases Search Screen - Advanced | 39,201 |
|  |  | my search terms | Search  Database - CINAHL Plus |  |
| S13 | ""Diarrheal disease*"" | Expanders - Apply equivalent subjects | Interface - EBSCOhost Research Databases | 910 |
|  |  | Search modes - Find all my search terms | Search Screen - Advanced Search  Database - CINAHL Plus |  |
| S12 | "Diarrhoea" | Expanders - Apply | Interface - EBSCOhost | 9,922 |
|  |  | equivalent subjects Search modes - Find all my search terms | Research Databases Search Screen - Advanced Search  Database - CINAHL Plus |  |
| S11 | (MH "Diarrhea") OR "Diarrhea" | Expanders - Apply equivalent subjects Search modes - Find all my search terms | Interface - EBSCOhost Research Databases Search Screen - Advanced Search | 21,473 |
|  |  |  | Database - CINAHL Plus |  |
| S10 | S1 OR S2 OR S3 OR S4 OR S5 OR S6 OR S7 OR S8 OR S9 | Expanders - Apply equivalent subjects Search modes - Find all | Interface - EBSCOhost Research Databases Search Screen - Advanced | 491,730 |
|  |  | my search terms | Search  Database - CINAHL Plus |  |
| S9 | "Newborn" | Expanders - Apply equivalent subjects | Interface - EBSCOhost Research Databases | 157,809 |

|  |  | Search modes - Find all my search terms | Search Screen - Advanced Search  Database - CINAHL Plus |  |
| --- | --- | --- | --- | --- |
| S8 | (MH "Child, Preschool") OR ""Child, preschool"" | Expanders - Apply equivalent subjects Search modes - Find all | Interface - EBSCOhost Research Databases Search Screen - Advanced | 232,149 |
|  |  | my search terms | Search  Database - CINAHL Plus |  |
| S7 | "Babies" | Expanders - Apply equivalent subjects | Interface - EBSCOhost Research Databases | 17,686 |
|  |  | Search modes - Find all my search terms | Search Screen - Advanced Search  Database - CINAHL Plus |  |
| S6 | (MH "Infant, Newborn+") | Expanders - Apply | Interface - EBSCOhost | 166,438 |
|  | OR "Neonate*" | equivalent subjects Search modes - Find all my search terms | Research Databases Search Screen - Advanced Search  Database - CINAHL Plus |  |
| S5 | (MH "Infant+") OR "Infant*" | Expanders - Apply equivalent subjects Search modes - Find all my search terms | Interface - EBSCOhost Research Databases Search Screen - Advanced Search | 342,454 |
|  |  |  | Database - CINAHL Plus |  |
| S4 | ""Under five*"" | Expanders - Apply equivalent subjects Search modes - Find all | Interface - EBSCOhost Research Databases Search Screen - Advanced | 18,550 |
|  |  | my search terms | Search  Database - CINAHL Plus |  |
| S3 | ""Under-five*"" | Expanders - Apply equivalent subjects | Interface - EBSCOhost Research Databases | 2,679 |
|  |  | Search modes - Find all my search terms | Search Screen - Advanced Search  Database - CINAHL Plus |  |
| S2 | ""Under five children"" | Expanders - Apply | Interface - EBSCOhost | 11,325 |
|  |  | equivalent subjects Search modes - Find all my search terms | Research Databases Search Screen - Advanced Search  Database - CINAHL Plus |  |
| S1 | ""Children under the age of five years"" | Expanders - Apply equivalent subjects Search modes - Find all my search terms | Interface - EBSCOhost Research Databases Search Screen - Advanced | 5,238 |

**PubMed search results**

| Search number | Query | Sort By | Filters | Search Details | Results | Time |
| --- | --- | --- | --- | --- | --- | --- |
| 71 | (((((("Malawi"[Mesh]) OR (Malawi)) OR ("Kenya"[Mesh])) OR (Kenya)) OR ("Ethiopia"[Mesh])) OR (Ethiopia)) AND ((((((((((((((((((((((("Disease burden") OR ("Burden of disease")) OR ("Hospitalization"[Mesh])) OR (Hospitalisation)) OR ("Inpatients"[Mesh])) OR (Inpatient*)) OR ("Outpatients"[Mesh])) OR (Outpatient*)) OR (("Epidemiology"[Mesh]) OR "epidemiology" [Subheading])) OR (Epidemiology)) OR ("Prevalence"[Mesh])) OR (Prevalence)) OR ("Incidence"[Mesh])) OR (Incidence)) OR ("Morbidity"[Mesh])) OR (Morbidity)) OR ("Mortality"[Mesh] OR "mortality" [Subheading])) OR (Mortality)) OR (Illness)) OR (Sickness)) OR ("Death"[Mesh])) OR (Death)) AND ((((((((((((("Children under the age of five years") OR (Under-five*)) OR ("Under five")) OR ("Infant"[Mesh])) OR (Infant*)) OR ("Infant, Newborn"[Mesh])) OR (Newborn*)) OR (Neonate*)) OR (Babies)) OR ("Child, Preschool"[Mesh])) OR ("Pre-school Child*")) OR (Under-5)) AND (((((((((((((((((((("Diarrhea"[Mesh]) OR (Diarrh*)) OR ("Gastroenteritis"[Mesh])) OR (Gastroenteritis)) OR ("Gastrointestinal infection*")) OR ("Gastro-intestinal infection*")) OR ("Rotavirus"[Mesh])) OR (Rotavirus)) OR ("Shigella"[Mesh])) OR (Shigella)) OR ("Dysentery, Bacillary"[Mesh])) OR ("Bacillary dysentery")) OR (Shigellosis)) OR ("Enterotoxigenic Escherichia coli"[Mesh])) OR ("Enterotoxigenic escherichia coli")) OR ("Escherichia coli Infections"[Mesh])) OR ("Escherichia coli Infection*")) OR ("Escherichia coli"[Mesh])) OR ("Escherichia coli")) OR (ETEC)))) |  | from 2000 - 2023 | (("Malawi"[MeSH Terms] OR ("Malawi"[MeSH Terms] OR "Malawi"[All Fields] OR "malawi s"[All Fields]) OR "Kenya"[MeSH Terms] OR ("Kenya"[MeSH Terms] OR "Kenya"[All Fields] OR "kenya s"[All Fields]) OR "Ethiopia"[MeSH Terms] OR ("Ethiopia"[MeSH Terms] OR "Ethiopia"[All Fields] OR "ethiopia s"[All Fields])) AND (("Disease burden"[All Fields] OR "Burden of disease"[All Fields] OR "Hospitalization"[MeSH Terms] OR ("hospital s"[All Fields] OR "hospitalisation"[All Fields] OR "Hospitalization"[MeSH Terms] OR "Hospitalization"[All Fields] OR "hospitalised"[All Fields] OR "hospitalising"[All Fields] OR "hospitality"[All Fields] OR "hospitalisations"[All Fields] OR "hospitalizations"[All Fields] OR "hospitalize"[All Fields] OR "hospitalized"[All Fields] OR "hospitalizing"[All Fields] OR "hospitals"[MeSH Terms] OR "hospitals"[All Fields] OR "hospital"[All Fields]) OR "Inpatients"[MeSH Terms] OR "inpatient*"[All Fields] OR "Outpatients"[MeSH Terms] OR "outpatient*"[All Fields] OR ("Epidemiology"[MeSH Terms] OR "Epidemiology"[MeSH Subheading]) OR ("epidemiologies"[All Fields] OR "Epidemiology"[MeSH Subheading] OR "Epidemiology"[All Fields] OR "Epidemiology"[MeSH Terms] OR "epidemiology s"[All Fields]) OR "Prevalence"[MeSH Terms] OR ("Epidemiology"[MeSH Subheading] OR "Epidemiology"[All Fields] OR "Prevalence"[All Fields] OR "Prevalence"[MeSH Terms] OR "prevalance"[All Fields] OR "prevalences"[All Fields] OR "prevalence s"[All Fields] OR "prevalent"[All Fields] OR "prevalently"[All Fields] OR "prevalents"[All Fields]) OR "Incidence"[MeSH Terms] OR ("Epidemiology"[MeSH Subheading] OR "Epidemiology"[All Fields] OR "Incidence"[All Fields] OR "Incidence"[MeSH Terms] OR "incidences"[All Fields] OR "incident"[All Fields] OR "incidents"[All Fields]) OR "Morbidity"[MeSH Terms] OR ("Epidemiology"[MeSH Subheading] OR "Epidemiology"[All Fields] OR "Morbidity"[All Fields] OR "Morbidity"[MeSH Terms] OR "morbid"[All Fields] OR "morbidities"[All Fields] OR "morbids"[All Fields]) OR ("Mortality"[MeSH Terms] OR "Mortality"[MeSH Subheading]) OR ("Mortality"[MeSH Terms] OR "Mortality"[All Fields] OR "mortalities"[All Fields] OR "Mortality"[MeSH Subheading]) OR ("illness"[All Fields] OR "illness s"[All Fields] OR "illnesses"[All Fields]) OR ("sickness"[All Fields] OR "sicknesses"[All Fields]) OR "Death"[MeSH Terms] OR ("Death"[MeSH Terms] OR "Death"[All Fields] OR "deaths"[All Fields])) AND (((("child"[MeSH Terms] OR "child"[All Fields] OR "children"[All Fields] OR "child s"[All Fields] OR "children s"[All Fields] OR "childrens"[All Fields] OR "childs"[All Fields]) AND "under"[All Fields] AND ("age"[Journal] OR ("the"[All Fields] AND "age"[All Fields]) OR "the age"[All Fields]) AND "five"[All Fields] AND "years"[All Fields]) OR "under five*"[All Fields] OR "Under five"[All Fields] OR "Infant"[MeSH Terms] OR "infant*"[All Fields] OR "infant, newborn"[MeSH Terms] OR "newborn*"[All Fields] OR "neonate*"[All Fields] OR ("baby s"[All Fields] OR "babys"[All Fields] OR "Infant"[MeSH Terms] OR "Infant"[All Fields] OR "babies"[All Fields]) OR "child, preschool"[MeSH Terms] OR "pre school child*"[All Fields] OR "Under-5"[All Fields]) AND ("Diarrhea"[MeSH Terms] OR "diarrh*"[All Fields] OR "Gastroenteritis"[MeSH Terms] OR ("gastroenteric"[All Fields] OR "Gastroenteritis"[MeSH Terms] OR "Gastroenteritis"[All Fields] OR "gastroenteritides"[All Fields]) OR "gastrointestinal infection*"[All Fields] OR "gastro intestinal infection*"[All Fields] OR "Rotavirus"[MeSH Terms] OR ("Rotavirus"[MeSH Terms] OR "Rotavirus"[All Fields] OR "rotaviruses"[All Fields]) OR "Shigella"[MeSH Terms] OR ("Shigella"[MeSH Terms] OR "Shigella"[All Fields] OR "shigellas"[All Fields] OR "dysentery, bacillary"[MeSH Terms] OR ("dysentery"[All Fields] AND "bacillary"[All Fields]) OR "Bacillary dysentery"[All Fields] OR "shigella s"[All Fields] OR "shigellae"[All Fields]) OR "dysentery, bacillary"[MeSH Terms] OR "Bacillary dysentery"[All Fields] OR ("dysentery, bacillary"[MeSH Terms] OR ("dysentery"[All Fields] AND "bacillary"[All Fields]) OR "Bacillary dysentery"[All Fields] OR "shigellosis"[All Fields]) OR "enterotoxigenic escherichia coli"[MeSH Terms] OR "enterotoxigenic escherichia coli"[All Fields] OR "Escherichia coli Infections"[MeSH Terms] OR "escherichia coli infection*"[All Fields] OR "Escherichia coli"[MeSH Terms] OR "Escherichia coli"[All Fields] OR ("enterotoxigenic escherichia coli"[MeSH Terms] OR ("enterotoxigenic"[All Fields] AND "escherichia"[All Fields] AND "coli"[All Fields]) OR "enterotoxigenic escherichia coli"[All Fields] OR "etec"[All Fields]))))) AND (2000:2023[pdat]) | 1,250 | 13:53:50 |
| 70 | (((((("Malawi"[Mesh]) OR (Malawi)) OR ("Kenya"[Mesh])) OR (Kenya)) OR ("Ethiopia"[Mesh])) OR (Ethiopia)) AND ((((((((((((((((((((((("Disease burden") OR ("Burden of disease")) OR ("Hospitalization"[Mesh])) OR (Hospitalisation)) OR ("Inpatients"[Mesh])) OR (Inpatient*)) OR ("Outpatients"[Mesh])) OR (Outpatient*)) OR (("Epidemiology"[Mesh]) OR "epidemiology" [Subheading])) OR (Epidemiology)) OR ("Prevalence"[Mesh])) OR (Prevalence)) OR ("Incidence"[Mesh])) OR (Incidence)) OR ("Morbidity"[Mesh])) OR (Morbidity)) OR ("Mortality"[Mesh] OR "mortality" [Subheading])) OR (Mortality)) OR (Illness)) OR (Sickness)) OR ("Death"[Mesh])) OR (Death)) AND ((((((((((((("Children under the age of five years") OR (Under-five*)) OR ("Under five")) OR ("Infant"[Mesh])) OR (Infant*)) OR ("Infant, Newborn"[Mesh])) OR (Newborn*)) OR (Neonate*)) OR (Babies)) OR ("Child, Preschool"[Mesh])) OR ("Pre-school Child*")) OR (Under-5)) AND (((((((((((((((((((("Diarrhea"[Mesh]) OR (Diarrh*)) OR ("Gastroenteritis"[Mesh])) OR (Gastroenteritis)) OR ("Gastrointestinal infection*")) OR ("Gastro-intestinal infection*")) OR ("Rotavirus"[Mesh])) OR (Rotavirus)) OR ("Shigella"[Mesh])) OR (Shigella)) OR ("Dysentery, Bacillary"[Mesh])) OR ("Bacillary dysentery")) OR (Shigellosis)) OR ("Enterotoxigenic Escherichia coli"[Mesh])) OR ("Enterotoxigenic escherichia coli")) OR ("Escherichia coli Infections"[Mesh])) OR ("Escherichia coli Infection*")) OR ("Escherichia coli"[Mesh])) OR ("Escherichia coli")) OR (ETEC)))) |  |  | ("Malawi"[MeSH Terms] OR ("Malawi"[MeSH Terms] OR "Malawi"[All Fields] OR "malawi s"[All Fields]) OR "Kenya"[MeSH Terms] OR ("Kenya"[MeSH Terms] OR "Kenya"[All Fields] OR "kenya s"[All Fields]) OR "Ethiopia"[MeSH Terms] OR ("Ethiopia"[MeSH Terms] OR "Ethiopia"[All Fields] OR "ethiopia s"[All Fields])) AND (("Disease burden"[All Fields] OR "Burden of disease"[All Fields] OR "Hospitalization"[MeSH Terms] OR ("hospital s"[All Fields] OR "hospitalisation"[All Fields] OR "Hospitalization"[MeSH Terms] OR "Hospitalization"[All Fields] OR "hospitalised"[All Fields] OR "hospitalising"[All Fields] OR "hospitality"[All Fields] OR "hospitalisations"[All Fields] OR "hospitalizations"[All Fields] OR "hospitalize"[All Fields] OR "hospitalized"[All Fields] OR "hospitalizing"[All Fields] OR "hospitals"[MeSH Terms] OR "hospitals"[All Fields] OR "hospital"[All Fields]) OR "Inpatients"[MeSH Terms] OR "inpatient*"[All Fields] OR "Outpatients"[MeSH Terms] OR "outpatient*"[All Fields] OR ("Epidemiology"[MeSH Terms] OR "Epidemiology"[MeSH Subheading]) OR ("epidemiologies"[All Fields] OR "Epidemiology"[MeSH Subheading] OR "Epidemiology"[All Fields] OR "Epidemiology"[MeSH Terms] OR "epidemiology s"[All Fields]) OR "Prevalence"[MeSH Terms] OR ("Epidemiology"[MeSH Subheading] OR "Epidemiology"[All Fields] OR "Prevalence"[All Fields] OR "Prevalence"[MeSH Terms] OR "prevalance"[All Fields] OR "prevalences"[All Fields] OR "prevalence s"[All Fields] OR "prevalent"[All Fields] OR "prevalently"[All Fields] OR "prevalents"[All Fields]) OR "Incidence"[MeSH Terms] OR ("Epidemiology"[MeSH Subheading] OR "Epidemiology"[All Fields] OR "Incidence"[All Fields] OR "Incidence"[MeSH Terms] OR "incidences"[All Fields] OR "incident"[All Fields] OR "incidents"[All Fields]) OR "Morbidity"[MeSH Terms] OR ("Epidemiology"[MeSH Subheading] OR "Epidemiology"[All Fields] OR "Morbidity"[All Fields] OR "Morbidity"[MeSH Terms] OR "morbid"[All Fields] OR "morbidities"[All Fields] OR "morbids"[All Fields]) OR ("Mortality"[MeSH Terms] OR "Mortality"[MeSH Subheading]) OR ("Mortality"[MeSH Terms] OR "Mortality"[All Fields] OR "mortalities"[All Fields] OR "Mortality"[MeSH Subheading]) OR ("illness"[All Fields] OR "illness s"[All Fields] OR "illnesses"[All Fields]) OR ("sickness"[All Fields] OR "sicknesses"[All Fields]) OR "Death"[MeSH Terms] OR ("Death"[MeSH Terms] OR "Death"[All Fields] OR "deaths"[All Fields])) AND (((("child"[MeSH Terms] OR "child"[All Fields] OR "children"[All Fields] OR "child s"[All Fields] OR "children s"[All Fields] OR "childrens"[All Fields] OR "childs"[All Fields]) AND "under"[All Fields] AND ("age"[Journal] OR ("the"[All Fields] AND "age"[All Fields]) OR "the age"[All Fields]) AND "five"[All Fields] AND "years"[All Fields]) OR "under five*"[All Fields] OR "Under five"[All Fields] OR "Infant"[MeSH Terms] OR "infant*"[All Fields] OR "infant, newborn"[MeSH Terms] OR "newborn*"[All Fields] OR "neonate*"[All Fields] OR ("baby s"[All Fields] OR "babys"[All Fields] OR "Infant"[MeSH Terms] OR "Infant"[All Fields] OR "babies"[All Fields]) OR "child, preschool"[MeSH Terms] OR "pre school child*"[All Fields] OR "Under-5"[All Fields]) AND ("Diarrhea"[MeSH Terms] OR "diarrh*"[All Fields] OR "Gastroenteritis"[MeSH Terms] OR ("gastroenteric"[All Fields] OR "Gastroenteritis"[MeSH Terms] OR "Gastroenteritis"[All Fields] OR "gastroenteritides"[All Fields]) OR "gastrointestinal infection*"[All Fields] OR "gastro intestinal infection*"[All Fields] OR "Rotavirus"[MeSH Terms] OR ("Rotavirus"[MeSH Terms] OR "Rotavirus"[All Fields] OR "rotaviruses"[All Fields]) OR "Shigella"[MeSH Terms] OR ("Shigella"[MeSH Terms] OR "Shigella"[All Fields] OR "shigellas"[All Fields] OR "dysentery, bacillary"[MeSH Terms] OR ("dysentery"[All Fields] AND "bacillary"[All Fields]) OR "Bacillary dysentery"[All Fields] OR "shigella s"[All Fields] OR "shigellae"[All Fields]) OR "dysentery, bacillary"[MeSH Terms] OR "Bacillary dysentery"[All Fields] OR ("dysentery, bacillary"[MeSH Terms] OR ("dysentery"[All Fields] AND "bacillary"[All Fields]) OR "Bacillary dysentery"[All Fields] OR "shigellosis"[All Fields]) OR "enterotoxigenic escherichia coli"[MeSH Terms] OR "enterotoxigenic escherichia coli"[All Fields] OR "Escherichia coli Infections"[MeSH Terms] OR "escherichia coli infection*"[All Fields] OR "Escherichia coli"[MeSH Terms] OR "Escherichia coli"[All Fields] OR ("enterotoxigenic escherichia coli"[MeSH Terms] OR ("enterotoxigenic"[All Fields] AND "escherichia"[All Fields] AND "coli"[All Fields]) OR "enterotoxigenic escherichia coli"[All Fields] OR "etec"[All Fields])))) | 1,459 | 13:53:35 |
| 69 | ((((("Malawi"[Mesh]) OR (Malawi)) OR ("Kenya"[Mesh])) OR (Kenya)) OR ("Ethiopia"[Mesh])) OR (Ethiopia) |  |  | "Malawi"[MeSH Terms] OR "Malawi"[MeSH Terms] OR "Malawi"[All Fields] OR "malawi s"[All Fields] OR "Kenya"[MeSH Terms] OR "Kenya"[MeSH Terms] OR "Kenya"[All Fields] OR "kenya s"[All Fields] OR "Ethiopia"[MeSH Terms] OR "Ethiopia"[MeSH Terms] OR "Ethiopia"[All Fields] OR "ethiopia s"[All Fields] | 88,963 | 13:53:17 |
| 68 | Ethiopia |  |  | "ethiopia"[MeSH Terms] OR "ethiopia"[All Fields] OR "ethiopia s"[All Fields] | 38,683 | 13:52:49 |
| 67 | "Ethiopia"[Mesh] | Most Recent |  | "Ethiopia"[MeSH Terms] | 19,304 | 13:52:37 |
| 66 | Kenya |  |  | "kenya"[MeSH Terms] OR "kenya"[All Fields] OR "kenya s"[All Fields] | 40,703 | 13:52:04 |
| 65 | "Kenya"[Mesh] | Most Recent |  | "Kenya"[MeSH Terms] | 19,478 | 13:51:53 |
| 64 | Malawi |  |  | "malawi"[MeSH Terms] OR "malawi"[All Fields] OR "malawi s"[All Fields] | 12,844 | 13:51:28 |
| 63 | "Malawi"[Mesh] | Most Recent |  | "Malawi"[MeSH Terms] | 6,719 | 13:51:15 |
| 62 | (((((((((((((((((((((("Disease burden") OR ("Burden of disease")) OR ("Hospitalization"[Mesh])) OR (Hospitalisation)) OR ("Inpatients"[Mesh])) OR (Inpatient*)) OR ("Outpatients"[Mesh])) OR (Outpatient*)) OR (("Epidemiology"[Mesh]) OR "epidemiology" [Subheading])) OR (Epidemiology)) OR ("Prevalence"[Mesh])) OR (Prevalence)) OR ("Incidence"[Mesh])) OR (Incidence)) OR ("Morbidity"[Mesh])) OR (Morbidity)) OR ("Mortality"[Mesh] OR "mortality" [Subheading])) OR (Mortality)) OR (Illness)) OR (Sickness)) OR ("Death"[Mesh])) OR (Death)) AND ((((((((((((("Children under the age of five years") OR (Under-five*)) OR ("Under five")) OR ("Infant"[Mesh])) OR (Infant*)) OR ("Infant, Newborn"[Mesh])) OR (Newborn*)) OR (Neonate*)) OR (Babies)) OR ("Child, Preschool"[Mesh])) OR ("Pre-school Child*")) OR (Under-5)) AND (((((((((((((((((((("Diarrhea"[Mesh]) OR (Diarrh*)) OR ("Gastroenteritis"[Mesh])) OR (Gastroenteritis)) OR ("Gastrointestinal infection*")) OR ("Gastro-intestinal infection*")) OR ("Rotavirus"[Mesh])) OR (Rotavirus)) OR ("Shigella"[Mesh])) OR (Shigella)) OR ("Dysentery, Bacillary"[Mesh])) OR ("Bacillary dysentery")) OR (Shigellosis)) OR ("Enterotoxigenic Escherichia coli"[Mesh])) OR ("Enterotoxigenic escherichia coli")) OR ("Escherichia coli Infections"[Mesh])) OR ("Escherichia coli Infection*")) OR ("Escherichia coli"[Mesh])) OR ("Escherichia coli")) OR (ETEC))) |  |  | ("Disease burden"[All Fields] OR "Burden of disease"[All Fields] OR "Hospitalization"[MeSH Terms] OR ("hospital s"[All Fields] OR "hospitalisation"[All Fields] OR "Hospitalization"[MeSH Terms] OR "Hospitalization"[All Fields] OR "hospitalised"[All Fields] OR "hospitalising"[All Fields] OR "hospitality"[All Fields] OR "hospitalisations"[All Fields] OR "hospitalizations"[All Fields] OR "hospitalize"[All Fields] OR "hospitalized"[All Fields] OR "hospitalizing"[All Fields] OR "hospitals"[MeSH Terms] OR "hospitals"[All Fields] OR "hospital"[All Fields]) OR "Inpatients"[MeSH Terms] OR "inpatient*"[All Fields] OR "Outpatients"[MeSH Terms] OR "outpatient*"[All Fields] OR ("Epidemiology"[MeSH Terms] OR "Epidemiology"[MeSH Subheading]) OR ("epidemiologies"[All Fields] OR "Epidemiology"[MeSH Subheading] OR "Epidemiology"[All Fields] OR "Epidemiology"[MeSH Terms] OR "epidemiology s"[All Fields]) OR "Prevalence"[MeSH Terms] OR ("Epidemiology"[MeSH Subheading] OR "Epidemiology"[All Fields] OR "Prevalence"[All Fields] OR "Prevalence"[MeSH Terms] OR "prevalance"[All Fields] OR "prevalences"[All Fields] OR "prevalence s"[All Fields] OR "prevalent"[All Fields] OR "prevalently"[All Fields] OR "prevalents"[All Fields]) OR "Incidence"[MeSH Terms] OR ("Epidemiology"[MeSH Subheading] OR "Epidemiology"[All Fields] OR "Incidence"[All Fields] OR "Incidence"[MeSH Terms] OR "incidences"[All Fields] OR "incident"[All Fields] OR "incidents"[All Fields]) OR "Morbidity"[MeSH Terms] OR ("Epidemiology"[MeSH Subheading] OR "Epidemiology"[All Fields] OR "Morbidity"[All Fields] OR "Morbidity"[MeSH Terms] OR "morbid"[All Fields] OR "morbidities"[All Fields] OR "morbids"[All Fields]) OR ("Mortality"[MeSH Terms] OR "Mortality"[MeSH Subheading]) OR ("Mortality"[MeSH Terms] OR "Mortality"[All Fields] OR "mortalities"[All Fields] OR "Mortality"[MeSH Subheading]) OR ("illness"[All Fields] OR "illness s"[All Fields] OR "illnesses"[All Fields]) OR ("sickness"[All Fields] OR "sicknesses"[All Fields]) OR "Death"[MeSH Terms] OR ("Death"[MeSH Terms] OR "Death"[All Fields] OR "deaths"[All Fields])) AND (((("child"[MeSH Terms] OR "child"[All Fields] OR "children"[All Fields] OR "child s"[All Fields] OR "children s"[All Fields] OR "childrens"[All Fields] OR "childs"[All Fields]) AND "under"[All Fields] AND ("age"[Journal] OR ("the"[All Fields] AND "age"[All Fields]) OR "the age"[All Fields]) AND "five"[All Fields] AND "years"[All Fields]) OR "under five*"[All Fields] OR "Under five"[All Fields] OR "Infant"[MeSH Terms] OR "infant*"[All Fields] OR "infant, newborn"[MeSH Terms] OR "newborn*"[All Fields] OR "neonate*"[All Fields] OR ("baby s"[All Fields] OR "babys"[All Fields] OR "Infant"[MeSH Terms] OR "Infant"[All Fields] OR "babies"[All Fields]) OR "child, preschool"[MeSH Terms] OR "pre school child*"[All Fields] OR "Under-5"[All Fields]) AND ("Diarrhea"[MeSH Terms] OR "diarrh*"[All Fields] OR "Gastroenteritis"[MeSH Terms] OR ("gastroenteric"[All Fields] OR "Gastroenteritis"[MeSH Terms] OR "Gastroenteritis"[All Fields] OR "gastroenteritides"[All Fields]) OR "gastrointestinal infection*"[All Fields] OR "gastro intestinal infection*"[All Fields] OR "Rotavirus"[MeSH Terms] OR ("Rotavirus"[MeSH Terms] OR "Rotavirus"[All Fields] OR "rotaviruses"[All Fields]) OR "Shigella"[MeSH Terms] OR ("Shigella"[MeSH Terms] OR "Shigella"[All Fields] OR "shigellas"[All Fields] OR "dysentery, bacillary"[MeSH Terms] OR ("dysentery"[All Fields] AND "bacillary"[All Fields]) OR "Bacillary dysentery"[All Fields] OR "shigella s"[All Fields] OR "shigellae"[All Fields]) OR "dysentery, bacillary"[MeSH Terms] OR "Bacillary dysentery"[All Fields] OR ("dysentery, bacillary"[MeSH Terms] OR ("dysentery"[All Fields] AND "bacillary"[All Fields]) OR "Bacillary dysentery"[All Fields] OR "shigellosis"[All Fields]) OR "enterotoxigenic escherichia coli"[MeSH Terms] OR "enterotoxigenic escherichia coli"[All Fields] OR "Escherichia coli Infections"[MeSH Terms] OR "escherichia coli infection*"[All Fields] OR "Escherichia coli"[MeSH Terms] OR "Escherichia coli"[All Fields] OR ("enterotoxigenic escherichia coli"[MeSH Terms] OR ("enterotoxigenic"[All Fields] AND "escherichia"[All Fields] AND "coli"[All Fields]) OR "enterotoxigenic escherichia coli"[All Fields] OR "etec"[All Fields]))) | 48,750 | 13:50:39 |
| 61 | ((((((((((((((((((((("Disease burden") OR ("Burden of disease")) OR ("Hospitalization"[Mesh])) OR (Hospitalisation)) OR ("Inpatients"[Mesh])) OR (Inpatient*)) OR ("Outpatients"[Mesh])) OR (Outpatient*)) OR (("Epidemiology"[Mesh]) OR "epidemiology" [Subheading])) OR (Epidemiology)) OR ("Prevalence"[Mesh])) OR (Prevalence)) OR ("Incidence"[Mesh])) OR (Incidence)) OR ("Morbidity"[Mesh])) OR (Morbidity)) OR ("Mortality"[Mesh] OR "mortality" [Subheading])) OR (Mortality)) OR (Illness)) OR (Sickness)) OR ("Death"[Mesh])) OR (Death) |  |  | "Disease burden"[All Fields] OR "Burden of disease"[All Fields] OR "Hospitalization"[MeSH Terms] OR "hospital s"[All Fields] OR "hospitalisation"[All Fields] OR "Hospitalization"[MeSH Terms] OR "Hospitalization"[All Fields] OR "hospitalised"[All Fields] OR "hospitalising"[All Fields] OR "hospitality"[All Fields] OR "hospitalisations"[All Fields] OR "hospitalizations"[All Fields] OR "hospitalize"[All Fields] OR "hospitalized"[All Fields] OR "hospitalizing"[All Fields] OR "hospitals"[MeSH Terms] OR "hospitals"[All Fields] OR "hospital"[All Fields] OR "Inpatients"[MeSH Terms] OR "inpatient*"[All Fields] OR "Outpatients"[MeSH Terms] OR "outpatient*"[All Fields] OR "Epidemiology"[MeSH Terms] OR "Epidemiology"[MeSH Subheading] OR "epidemiologies"[All Fields] OR "Epidemiology"[MeSH Subheading] OR "Epidemiology"[All Fields] OR "Epidemiology"[MeSH Terms] OR "epidemiology s"[All Fields] OR "Prevalence"[MeSH Terms] OR "Epidemiology"[MeSH Subheading] OR "Epidemiology"[All Fields] OR "Prevalence"[All Fields] OR "Prevalence"[MeSH Terms] OR "prevalance"[All Fields] OR "prevalences"[All Fields] OR "prevalence s"[All Fields] OR "prevalent"[All Fields] OR "prevalently"[All Fields] OR "prevalents"[All Fields] OR "Incidence"[MeSH Terms] OR "Epidemiology"[MeSH Subheading] OR "Epidemiology"[All Fields] OR "Incidence"[All Fields] OR "Incidence"[MeSH Terms] OR "incidences"[All Fields] OR "incident"[All Fields] OR "incidents"[All Fields] OR "Morbidity"[MeSH Terms] OR "Epidemiology"[MeSH Subheading] OR "Epidemiology"[All Fields] OR "Morbidity"[All Fields] OR "Morbidity"[MeSH Terms] OR "morbid"[All Fields] OR "morbidities"[All Fields] OR "morbids"[All Fields] OR "Mortality"[MeSH Terms] OR "Mortality"[MeSH Subheading] OR "Mortality"[MeSH Terms] OR "Mortality"[All Fields] OR "mortalities"[All Fields] OR "Mortality"[MeSH Subheading] OR "illness"[All Fields] OR "illness s"[All Fields] OR "illnesses"[All Fields] OR "sickness"[All Fields] OR "sicknesses"[All Fields] OR "Death"[MeSH Terms] OR "Death"[MeSH Terms] OR "Death"[All Fields] OR "deaths"[All Fields] | ######## | 13:50:05 |
| 60 | Death |  |  | "death"[MeSH Terms] OR "death"[All Fields] OR "deaths"[All Fields] | 1,196,745 | 13:47:56 |
| 59 | "Death"[Mesh] | Most Recent |  | "Death"[MeSH Terms] | 164,596 | 13:47:21 |
| 58 | Sickness |  |  | "sickness"[All Fields] OR "sicknesses"[All Fields] | 41,433 | 13:46:49 |
| 57 | Illness |  |  | "illness"[All Fields] OR "illness s"[All Fields] OR "illnesses"[All Fields] | 646,219 | 13:46:01 |
| 56 | Mortality |  |  | "mortality"[MeSH Terms] OR "mortality"[All Fields] OR "mortalities"[All Fields] OR "mortality"[MeSH Subheading] | 1,531,936 | 13:45:22 |
| 55 | "Mortality"[Mesh] OR "mortality" [Subheading] | Most Recent |  | "Mortality"[MeSH Terms] OR "Mortality"[MeSH Subheading] | 857,350 | 13:45:04 |
| 54 | Morbidity |  |  | "epidemiology"[MeSH Subheading] OR "epidemiology"[All Fields] OR "morbidity"[All Fields] OR "morbidity"[MeSH Terms] OR "morbid"[All Fields] OR "morbidities"[All Fields] OR "morbids"[All Fields] | 3,481,374 | 13:43:28 |
| 53 | "Morbidity"[Mesh] | Most Recent |  | "Morbidity"[MeSH Terms] | 649,509 | 13:43:16 |
| 52 | Incidence |  |  | "epidemiology"[MeSH Subheading] OR "epidemiology"[All Fields] OR "incidence"[All Fields] OR "incidence"[MeSH Terms] OR "incidences"[All Fields] OR "incident"[All Fields] OR "incidents"[All Fields] | 3,693,503 | 13:42:48 |
| 51 | "Incidence"[Mesh] | Most Recent |  | "Incidence"[MeSH Terms] | 301,247 | 13:42:34 |
| 50 | Prevalence |  |  | "epidemiology"[MeSH Subheading] OR "epidemiology"[All Fields] OR "prevalence"[All Fields] OR "prevalence"[MeSH Terms] OR "prevalance"[All Fields] OR "prevalences"[All Fields] OR "prevalence s"[All Fields] OR "prevalent"[All Fields] OR "prevalently"[All Fields] OR "prevalents"[All Fields] | 3,562,484 | 13:42:10 |
| 49 | "Prevalence"[Mesh] | Most Recent |  | "Prevalence"[MeSH Terms] | 343,741 | 13:41:56 |
| 48 | Epidemiology |  |  | "epidemiologies"[All Fields] OR "epidemiology"[MeSH Subheading] OR "epidemiology"[All Fields] OR "epidemiology"[MeSH Terms] OR "epidemiology s"[All Fields] | 3,062,641 | 13:41:23 |
| 47 | ("Epidemiology"[Mesh]) OR "epidemiology" [Subheading] | Most Recent |  | "Epidemiology"[MeSH Terms] OR "Epidemiology"[MeSH Subheading] | 2,685,029 | 13:41:08 |
| 46 | Outpatient* |  |  | "outpatient*"[All Fields] | 248,284 | 13:40:01 |
| 45 | "Outpatients"[Mesh] | Most Recent |  | "Outpatients"[MeSH Terms] | 21,508 | 13:39:42 |
| 44 | Inpatient* |  |  | "inpatient*"[All Fields] | 164,632 | 13:39:02 |
| 43 | "Inpatients"[Mesh] | Most Recent |  | "Inpatients"[MeSH Terms] | 29,463 | 13:38:40 |
| 42 | Hospitalisation |  |  | "hospital s"[All Fields] OR "hospitalisation"[All Fields] OR "hospitalization"[MeSH Terms] OR "hospitalization"[All Fields] OR "hospitalised"[All Fields] OR "hospitalising"[All Fields] OR "hospitality"[All Fields] OR "hospitalisations"[All Fields] OR "hospitalizations"[All Fields] OR "hospitalize"[All Fields] OR "hospitalized"[All Fields] OR "hospitalizing"[All Fields] OR "hospitals"[MeSH Terms] OR "hospitals"[All Fields] OR "hospital"[All Fields] | 6,804,203 | 13:36:47 |
| 40 | "Hospitalization"[Mesh] | Most Recent |  | "Hospitalization"[MeSH Terms] | 292,274 | 13:35:31 |
| 39 | "Burden of disease" |  |  | "Burden of disease"[All Fields] | 15,581 | 13:34:31 |
| 38 | "Disease burden" |  |  | "Disease burden"[All Fields] | 22,495 | 13:34:15 |
| 37 | (((((((((((("Children under the age of five years") OR (Under-five*)) OR ("Under five")) OR ("Infant"[Mesh])) OR (Infant*)) OR ("Infant, Newborn"[Mesh])) OR (Newborn*)) OR (Neonate*)) OR (Babies)) OR ("Child, Preschool"[Mesh])) OR ("Pre-school Child*")) OR (Under-5)) AND (((((((((((((((((((("Diarrhea"[Mesh]) OR (Diarrh*)) OR ("Gastroenteritis"[Mesh])) OR (Gastroenteritis)) OR ("Gastrointestinal infection*")) OR ("Gastro-intestinal infection*")) OR ("Rotavirus"[Mesh])) OR (Rotavirus)) OR ("Shigella"[Mesh])) OR (Shigella)) OR ("Dysentery, Bacillary"[Mesh])) OR ("Bacillary dysentery")) OR (Shigellosis)) OR ("Enterotoxigenic Escherichia coli"[Mesh])) OR ("Enterotoxigenic escherichia coli")) OR ("Escherichia coli Infections"[Mesh])) OR ("Escherichia coli Infection*")) OR ("Escherichia coli"[Mesh])) OR ("Escherichia coli")) OR (ETEC)) |  |  | ((("child"[MeSH Terms] OR "child"[All Fields] OR "children"[All Fields] OR "child s"[All Fields] OR "children s"[All Fields] OR "childrens"[All Fields] OR "childs"[All Fields]) AND "under"[All Fields] AND ("age"[Journal] OR ("the"[All Fields] AND "age"[All Fields]) OR "the age"[All Fields]) AND "five"[All Fields] AND "years"[All Fields]) OR "under five*"[All Fields] OR "Under five"[All Fields] OR "Infant"[MeSH Terms] OR "infant*"[All Fields] OR "infant, newborn"[MeSH Terms] OR "newborn*"[All Fields] OR "neonate*"[All Fields] OR ("baby s"[All Fields] OR "babys"[All Fields] OR "Infant"[MeSH Terms] OR "Infant"[All Fields] OR "babies"[All Fields]) OR "child, preschool"[MeSH Terms] OR "pre school child*"[All Fields] OR "Under-5"[All Fields]) AND ("Diarrhea"[MeSH Terms] OR "diarrh*"[All Fields] OR "Gastroenteritis"[MeSH Terms] OR ("gastroenteric"[All Fields] OR "Gastroenteritis"[MeSH Terms] OR "Gastroenteritis"[All Fields] OR "gastroenteritides"[All Fields]) OR "gastrointestinal infection*"[All Fields] OR "gastro intestinal infection*"[All Fields] OR "Rotavirus"[MeSH Terms] OR ("Rotavirus"[MeSH Terms] OR "Rotavirus"[All Fields] OR "rotaviruses"[All Fields]) OR "Shigella"[MeSH Terms] OR ("Shigella"[MeSH Terms] OR "Shigella"[All Fields] OR "shigellas"[All Fields] OR "dysentery, bacillary"[MeSH Terms] OR ("dysentery"[All Fields] AND "bacillary"[All Fields]) OR "Bacillary dysentery"[All Fields] OR "shigella s"[All Fields] OR "shigellae"[All Fields]) OR "dysentery, bacillary"[MeSH Terms] OR "Bacillary dysentery"[All Fields] OR ("dysentery, bacillary"[MeSH Terms] OR ("dysentery"[All Fields] AND "bacillary"[All Fields]) OR "Bacillary dysentery"[All Fields] OR "shigellosis"[All Fields]) OR "enterotoxigenic escherichia coli"[MeSH Terms] OR "enterotoxigenic escherichia coli"[All Fields] OR "Escherichia coli Infections"[MeSH Terms] OR "escherichia coli infection*"[All Fields] OR "Escherichia coli"[MeSH Terms] OR "Escherichia coli"[All Fields] OR ("enterotoxigenic escherichia coli"[MeSH Terms] OR ("enterotoxigenic"[All Fields] AND "escherichia"[All Fields] AND "coli"[All Fields]) OR "enterotoxigenic escherichia coli"[All Fields] OR "etec"[All Fields])) | 77,964 | 13:28:11 |
| 36 | ((((((((((((((((((("Diarrhea"[Mesh]) OR (Diarrh*)) OR ("Gastroenteritis"[Mesh])) OR (Gastroenteritis)) OR ("Gastrointestinal infection*")) OR ("Gastro-intestinal infection*")) OR ("Rotavirus"[Mesh])) OR (Rotavirus)) OR ("Shigella"[Mesh])) OR (Shigella)) OR ("Dysentery, Bacillary"[Mesh])) OR ("Bacillary dysentery")) OR (Shigellosis)) OR ("Enterotoxigenic Escherichia coli"[Mesh])) OR ("Enterotoxigenic escherichia coli")) OR ("Escherichia coli Infections"[Mesh])) OR ("Escherichia coli Infection*")) OR ("Escherichia coli"[Mesh])) OR ("Escherichia coli")) OR (ETEC) |  |  | "Diarrhea"[MeSH Terms] OR "diarrh*"[All Fields] OR "Gastroenteritis"[MeSH Terms] OR ("gastroenteric"[All Fields] OR "Gastroenteritis"[MeSH Terms] OR "Gastroenteritis"[All Fields] OR "gastroenteritides"[All Fields]) OR "gastrointestinal infection*"[All Fields] OR "gastro intestinal infection*"[All Fields] OR "Rotavirus"[MeSH Terms] OR ("Rotavirus"[MeSH Terms] OR "Rotavirus"[All Fields] OR "rotaviruses"[All Fields]) OR "Shigella"[MeSH Terms] OR ("Shigella"[MeSH Terms] OR "Shigella"[All Fields] OR "shigellas"[All Fields] OR "dysentery, bacillary"[MeSH Terms] OR ("dysentery"[All Fields] AND "bacillary"[All Fields]) OR "Bacillary dysentery"[All Fields] OR "shigella s"[All Fields] OR "shigellae"[All Fields]) OR "dysentery, bacillary"[MeSH Terms] OR "Bacillary dysentery"[All Fields] OR ("dysentery, bacillary"[MeSH Terms] OR ("dysentery"[All Fields] AND "bacillary"[All Fields]) OR "Bacillary dysentery"[All Fields] OR "shigellosis"[All Fields]) OR "enterotoxigenic escherichia coli"[MeSH Terms] OR "enterotoxigenic escherichia coli"[All Fields] OR "Escherichia coli Infections"[MeSH Terms] OR "escherichia coli infection*"[All Fields] OR "Escherichia coli"[MeSH Terms] OR "Escherichia coli"[All Fields] OR ("enterotoxigenic escherichia coli"[MeSH Terms] OR ("enterotoxigenic"[All Fields] AND "escherichia"[All Fields] AND "coli"[All Fields]) OR "enterotoxigenic escherichia coli"[All Fields] OR "etec"[All Fields]) | 812,137 | 13:27:48 |
| 35 | ETEC |  |  | "enterotoxigenic escherichia coli"[MeSH Terms] OR ("enterotoxigenic"[All Fields] AND "escherichia"[All Fields] AND "coli"[All Fields]) OR "enterotoxigenic escherichia coli"[All Fields] OR "etec"[All Fields] | 5,311 | 13:25:32 |
| 34 | "Escherichia coli" |  |  | "Escherichia coli"[All Fields] | 426,554 | 13:24:57 |
| 33 | "Escherichia coli"[Mesh] | Most Recent |  | "Escherichia coli"[MeSH Terms] | 304,284 | 13:24:29 |
| 32 | "Escherichia coli Infection*" |  |  | "escherichia coli infection*"[All Fields] | 36,002 | 13:23:10 |
| 31 | "Escherichia coli Infections"[Mesh] | Most Recent |  | "Escherichia coli Infections"[MeSH Terms] | 35,656 | 13:22:51 |
| 30 | "Enterotoxigenic escherichia coli" |  |  | "Enterotoxigenic escherichia coli"[All Fields] | 3,673 | 13:22:01 |
| 29 | "Enterotoxigenic Escherichia coli"[Mesh] | Most Recent |  | "Enterotoxigenic Escherichia coli"[MeSH Terms] | 1,273 | 13:21:48 |
| 28 | Shigellosis |  |  | "dysentery, bacillary"[MeSH Terms] OR ("dysentery"[All Fields] AND "bacillary"[All Fields]) OR "bacillary dysentery"[All Fields] OR "shigellosis"[All Fields] | 9,173 | 13:20:56 |
| 27 | "Bacillary dysentery" |  |  | "Bacillary dysentery"[All Fields] | 8,437 | 13:20:03 |
| 26 | "Dysentery, Bacillary"[Mesh] | Most Recent |  | "dysentery, bacillary"[MeSH Terms] | 8,090 | 13:19:39 |
| 25 | Shigella |  |  | "shigella"[MeSH Terms] OR "shigella"[All Fields] OR "shigellas"[All Fields] OR "dysentery, bacillary"[MeSH Terms] OR ("dysentery"[All Fields] AND "bacillary"[All Fields]) OR "bacillary dysentery"[All Fields] OR "shigella s"[All Fields] OR "shigellae"[All Fields] | 22,916 | 13:19:05 |
| 24 | "Shigella"[Mesh] | Most Recent |  | "Shigella"[MeSH Terms] | 12,344 | 13:18:52 |
| 23 | "Rotavirus infection*" |  |  | "rotavirus infection*"[All Fields] | 9,544 | 13:17:54 |
| 22 | "Rotavirus Infections"[Mesh] | Most Recent |  | "Rotavirus Infections"[MeSH Terms] | 8,709 | 13:17:19 |
| 21 | Rotavirus |  |  | "rotavirus"[MeSH Terms] OR "rotavirus"[All Fields] OR "rotaviruses"[All Fields] | 17,664 | 13:16:51 |
| 20 | "Rotavirus"[Mesh] | Most Recent |  | "Rotavirus"[MeSH Terms] | 9,831 | 13:16:35 |
| 19 | "Gastro-intestinal infection*" |  |  | "gastro intestinal infection*"[All Fields] | 107 | 13:15:52 |
| 18 | "Gastrointestinal infection*" |  |  | "gastrointestinal infection*"[All Fields] | 3,342 | 13:15:19 |
| 17 | Gastroenteritis |  |  | "gastroenteric"[All Fields] OR "gastroenteritis"[MeSH Terms] OR "gastroenteritis"[All Fields] OR "gastroenteritides"[All Fields] | 261,453 | 13:14:22 |
| 16 | "Gastroenteritis"[Mesh] | Most Recent |  | "Gastroenteritis"[MeSH Terms] | 248,261 | 13:13:59 |
| 15 | Diarrh* |  |  | "diarrh*"[All Fields] | 146,211 | 13:13:08 |
| 14 | "Diarrhea"[Mesh] | Most Recent |  | "Diarrhea"[MeSH Terms] | 57,640 | 13:12:52 |
| 13 | ((((((((((("Children under the age of five years") OR (Under-five*)) OR ("Under five")) OR ("Infant"[Mesh])) OR (Infant*)) OR ("Infant, Newborn"[Mesh])) OR (Newborn*)) OR (Neonate*)) OR (Babies)) OR ("Child, Preschool"[Mesh])) OR ("Pre-school Child*")) OR (Under-5) |  |  | (("child"[MeSH Terms] OR "child"[All Fields] OR "children"[All Fields] OR "child s"[All Fields] OR "children s"[All Fields] OR "childrens"[All Fields] OR "childs"[All Fields]) AND "under"[All Fields] AND ("age"[Journal] OR ("the"[All Fields] AND "age"[All Fields]) OR "the age"[All Fields]) AND "five"[All Fields] AND "years"[All Fields]) OR "under five*"[All Fields] OR "Under five"[All Fields] OR "Infant"[MeSH Terms] OR "infant*"[All Fields] OR "infant, newborn"[MeSH Terms] OR "newborn*"[All Fields] OR "neonate*"[All Fields] OR ("baby s"[All Fields] OR "babys"[All Fields] OR "Infant"[MeSH Terms] OR "Infant"[All Fields] OR "babies"[All Fields]) OR "child, preschool"[MeSH Terms] OR "pre school child*"[All Fields] OR "Under-5"[All Fields] | 2,076,243 | 13:08:55 |
| 12 | Under-5 |  |  | "Under-5"[All Fields] | 9,852 | 13:07:25 |
| 11 | "Pre-school Child*" |  |  | "pre school child*"[All Fields] | 3,185 | 13:06:13 |
| 10 | "Child, Preschool"[Mesh] | Most Recent |  | "child, preschool"[MeSH Terms] | 990,058 | 13:05:52 |
| 9 | Babies |  |  | "baby s"[All Fields] OR "babys"[All Fields] OR "infant"[MeSH Terms] OR "infant"[All Fields] OR "babies"[All Fields] | 1,343,468 | 13:05:11 |
| 8 | Neonate* |  |  | "neonate*"[All Fields] | 112,461 | 13:04:34 |
| 7 | Newborn* |  |  | "newborn*"[All Fields] | 842,660 | 13:03:45 |
| 6 | "Infant, Newborn"[Mesh] | Most Recent |  | "infant, newborn"[MeSH Terms] | 673,882 | 13:02:18 |
| 5 | Infant* |  |  | "infant*"[All Fields] | 1,430,702 | 13:01:24 |
| 4 | "Infant"[Mesh] | Most Recent |  | "Infant"[MeSH Terms] | 1,252,285 | 13:00:58 |
| 3 | "Under five" |  |  | "Under five"[All Fields] | 7,807 | 12:59:41 |
| 2 | Under-five* |  |  | "under five*"[All Fields] | 8,168 | 12:59:11 |
| 1 | "Children under the age of five years" |  |  | ("child"[MeSH Terms] OR "child"[All Fields] OR "children"[All Fields] OR "child s"[All Fields] OR "children s"[All Fields] OR "childrens"[All Fields] OR "childs"[All Fields]) AND "under"[All Fields] AND ("age"[Journal] OR ("the"[All Fields] AND "age"[All Fields]) OR "the age"[All Fields]) AND "five"[All Fields] AND "years"[All Fields] | 1,825 | 12:57:22 |
