# Supplementary material for: Burden of Shigella and enterotoxigenic Escherichia coli infections among children under 5 years in Ethiopia, Kenya and Malawi: a systematic review and meta-analysis
Source: BMJ Glob Health. 2026 Mar 2;11(3):e018515. doi: 10.1136/bmjgh-2024-018515 (PMC12958989; doi:10.1136/bmjgh-2024-018515)
Supplement: online supplemental file 2 [file bmjgh-11-3-s002.docx]

# **Coding manual**

**SUPPLEMENTARY APPENDIX 2: NEWCASTLE-OTTAWA SCALE ADAPTED FOR CROSS-SECTIONAL STUDIES**

***Note:*** This Scale includes 3 categories, with a maximum of 9 points, based on: Selection (maximum of 4 stars); Comparability (maximum of 2 stars); and Outcome (maximum of 3 stars).

A study can be awarded a maximum of one star for each numbered item within the Selection and

Exposure categories. A maximum of two stars can be given for Comparability.

**Selection: *(Maximum 4 stars)***

1) Representativeness of the sample:

1. Truly representative of the average in the target population. * (all subjects or random sampling)
2. Somewhat representative of the average in the target population. * (non-random sampling)
3. Selected group of users.
4. No description of the sampling strategy.

2) Sample size:

1. Justified and satisfactory. *
2. Not justified.

3) Non-respondents:

1. Comparability between respondents and non-respondents’ characteristics is established, and the response rate is satisfactory. *
2. The response rate is unsatisfactory, or the comparability between respondents and non-respondents is unsatisfactory.
3. No description of the response rate or the characteristics of the responders and the non-responders.

4) Ascertainment of the exposure (risk factor):

1. Validated measurement tool. *
2. Non-validated measurement tool, but the tool is available or described. *
3. No description of the measurement tool.

**Comparability: *(Maximum 2 stars)***

1) The subjects in different outcome groups are comparable, based on the study design or analysis. Confounding factors are controlled.

1. The study controls for the most important factor (select one). *
2. The study controls for any additional factor. *

**Outcome: *(Maximum 3 stars)***

1) Assessment of the outcome:

1. Independent blind assessment or validated by laboratory methods. **
2. Record linkage. *
3. Self-report.
4. No description.

2) Statistical test:

1. The statistical test used to analyze the data is clearly described and appropriate, and the measurement of the association is presented, including confidence intervals and the probability level (p value). *
2. The statistical test is not appropriate.

| Very Good Studies: | 9 points |
| --- | --- |
| Good Studies: | 7-8 points |
| Satisfactory Studies: | 5-6 points |
| Unsatisfactory Studies: | 0 to 4 points |

This scale has been adapted from the Newcastle-Ottawa Quality Assessment Scale for cohort and case-control studies to perform a quality assessment of cross-sectional studies for this systematic review. This scale was a modified version of the NOS scale, as also used by several other studies that have felt the need to adapt the NOS scale so as to appropriately assess the quality of cross-sectional studies.

**NEWCASTLE - OTTAWA QUALITY ASSESSMENT SCALE FOR CASE CONTROL STUDIES**

***Note:*** This Scale includes 3 categories, with a maximum of 9 points, based on: Selection (maximum of 4 stars); Comparability (maximum of 2 stars); and Exposure (maximum of 3 stars).

A study can be awarded a maximum of one star for each numbered item within the Selection and Exposure categories. A maximum of two stars can be given for Comparability.

**Selection: (maximum of 4 stars)**

1) Is the case definition adequate?

1. Yes, with independent validation*
2. Yes, e.g. record linkage or based on self-reports
3. No description

2) Representativeness of the cases

1. Consecutive or obviously representative series of cases*
2. Potential for selection biases or not stated

3) Selection of Controls

1. community controls*
2. hospital controls
3. no description

4) Definition of Controls

1. No history of disease (endpoint)*
2. No description of source

**Comparability: (maximum of 2 stars)**

1) Comparability of cases and controls on the basis of the design or analysis

1. Study controls for _______________ (Select the most important factor.) *
2. Study controls for any additional factor* (This criterion could be modified to indicate specific control for a second important factor.)

**Exposure (maximum of 3 stars)**

1) Ascertainment of exposure

1. Secure record (e.g. surgical records) *
2. Structured interview where blind to case/control status*
3. Interview not blinded to case/control status
4. Written self-report or medical record only
5. No description

2) Same method of ascertainment for cases and controls

1. Yes*
2. No

3) Non-Response rate

1. Same rate for both groups*
2. Non respondents described
3. Rate different and no designation

| **Quality rating** | **#Points in Selection domain** | **#Points in Comparability domain** | **#Points in Outcome domain** |
| --- | --- | --- | --- |
| **Good** | ≥3 | ≥2 | ≥2 |
| **Fair** | 2 | ≥1 | ≥2 |
| **Poor** | 0-1 | 0 | 0-1 |
