# Supplementary material for: Burden of Shigella and enterotoxigenic Escherichia coli infections among children under 5 years in Ethiopia, Kenya and Malawi: a systematic review and meta-analysis
Source: BMJ Glob Health. 2026 Mar 2;11(3):e018515. doi: 10.1136/bmjgh-2024-018515 (PMC12958989; doi:10.1136/bmjgh-2024-018515)
Supplement: online supplemental file 3 [file bmjgh-11-3-s003.docx]

**Supplementary appendix 3**

**Table 1: Description of *Shigella* and ETEC papers**

| **Study** | **Study design** | **Study area (region, district, city, or county)** | **Study setting** | **Population** | **Study pathogen(s)** | ***Shigella* Pathogen identification method(s)** | **ETEC identification**  **Method(s)** | **Sample size*** | ***Shigella* prevalence N (%)** | **ETEC prevalence N (%)** | **Quality score** |  |
| --- | --- | --- | --- | --- | --- | --- | --- | --- | --- | --- | --- | --- |
| **Ethiopia** | | | | | | | | | | | | |
| Abebe W. et al. (2018)^20^ | Cross-sectional | SNNP | Urban health facility | OCD | *Shigella* and *Salmonella* | Culture | NA | 204 | 17 (8.3%) | NA | 7 |  |
| Debas G. et al. (2011)^21^ | Cross-sectional | Amhara | Urban and rural health facility | OCD | *Shigella* | Culture | NA | 50 | 11 (22%) | NA | 6 |  |
| Dessale M. et al. (2023)^22^ | Cross-sectional | Amhara | Urban health facility | OCD | *Shigella* and *Salmonella* | Culture | NA | 222 | 19 (8.6%) | NA | 7 |  |
| Balew M. et al. (2023)^23^ | Cross-sectional | Amhara | Rural health facility | OCD | Multiple pathogens | Culture | NA | 196 | 8 (4%) | NA | 7 |  |
| Kahsay AG. et al. (2015)^24^ | Cross-sectional | Tigray | Urban health facility | OCD | *Shigella* | Culture | NA | 241 | 32 (13.3%) | NA | 7 |  |
| Getamesay M. et al. (2014)^25^ | Cross-sectional | SNNP | Urban health facilities | OCD | *Shigella* and *Salmonella* | Culture | NA | 158 | 11 (7%) | NA | 6 |  |
| Abera B. et al. (2020)^26^ | Cross-sectional | Amhara | Urban and rural health facilities | OCD | Multiple pathogens | Culture | NA | 344 | 4 (1.2%) | NA | 7 |  |
| Ameya G. et al. (2018)^27^ | Cross-sectional | Amhara | Urban and rural health facilities | OCD | *Shigella* and *Salmonella* | Culture | NA | 167 | 8 (5%) | NA | 7 |  |
| Feleke H. et al. (2018)^28^ | Cross-sectional | Amhara | Rural community | Community children with diarrhoea | Multiple pathogens | Culture | NA | 112 | 3 (3%) | NA | 7 |  |
| Zelelie T. et al. (2019)^29^ | Cross-sectional | Amhara | Urban health facilities | OCD | Multiple pathogens | Culture | NA | 163 | 3 (2%) | NA | 7 |  |
| Mekonnen GK. et al. (2019)^30^ | Case-control | Gambella | Rural health facility | OCD and controls without diarrhoea | Multiple pathogens | Culture | NA | 134 | 14 (10%) | NA | 7 |  |
| Gebreegziabher G. et al. (2018)^31^ | Cross-sectional | Tigray | Urban and rural health facilities | OCD | *Shigella* and *Salmonella* | Culture | NA | 115 | 18 (16%) | NA | 7 |  |
| Mulu W. et al. (2017)^32^ | Surveillance | Amhara | Urban health facility | Hospitalised children with diarrhoea | Multiple pathogens | Culture | NA | 50 | 2 (4%) | NA | 7 |  |
| Tosisa et al. (2020)^33^ | Cross-sectional | Oromia | Urban health facility | OCD | *Shigella* and *Salmonella* | Culture | NA | 239 | 6 (3%) | NA | 7 |  |
| Mekonnen et al. (2018)^34^ | Observational | Dire Dawa | Urban health facility | OCD | Multiple pathogens | Culture | NA | 196 | 11 (6%) | NA | 7 |  |
| Beyene et al. (2014)^35^ | Cross-sectional | Oromia | Rural health facility | OCD | *Shigella* and *Salmonella* | Culture | NA | 260 | 6 (2%) | NA | 6 |  |
| Mamuye et al. (2015)^36^ | Cross-sectional | Addis Ababa | Urban health facilities | OCD | *Shigella* and *Salmonella* | Culture | NA | 253 | 23 (9%) | NA | 7 |  |
| Ayele B. et al. (2023)^37^ | Cross-sectional | Addis Ababa | Urban health facilities | OCD | *Shigella* | Culture | NA | 534 | 47 (9%) | NA | 7 |  |
| Assefa et al. (2019)^38^ | Cross-sectional | Oromia | Urban health facility | OCD | *Shigella* and *Salmonella* | Culture | NA | 422 | 18 (4%) | NA | 7 |  |
| Admassu et al. (2015)^39^ | Cross-sectional | Amhara | Urban health facility | OCD | *Shigella* and *Salmonella* | Culture | NA | 422 | 40 (9%) | NA | 6 |  |
| **Kenya** | | | | | | | | | | | | |
| Leting et al. (2021)^40^ | Cross-sectional | Turkana County | Rural health facility | OCD | *Shigella* and *Salmonella* | Culture | NA | 196 | 18 (9%) | NA | 5 |  |
| Pavlinac et al. (2016)^41^ | Surveillance | Kisii, Homabay, and Migori counties | Urban and rural health facilities | OCD | Multiple pathogens | Culture | PCR | 1360 | 63 (4.6%) | 41 (3%) | 7 |  |
| Zachariah et al. (2021)^42^ | Cross-sectional | Nandi County | Urban health facility | Hospitalised children with diarrhoea | *Shigella* and *Camylobacter jejuni* | Culture | NA | 139 | 28 (20%) | NA | 7 |  |
| Sang et al. (2012)^43^ | Cross-sectional | Malindi, Busia, Kisumu, and Nairobi counties | Urban and rural health facilities | OCD | Multiple pathogens | Culture | PCR | 651 | 15 (2.3%) | 8 (1.2%) | 5 |  |
| Kasumba et al. (2023)^44^ | Case-control | Siaya county | Unspecified | OCD and controls without diarrhoea | *Shigella* | Culture | NA | 1554 | 130 (8.4%) | NA | 7 |  |
| Beatty et al. (2009)^45^ | Surveillance | Kisumu county | Urban and rural health facility | OCD | Multiple pathogens | Culture |  | 2550 | 116 (5%) |  | 7 |  |
| Swierczewski BE. et al. (2013)^46^ | Case-control | Kericho and Kisumu counties | Urban and rural health facilities | OCD and controls without diarrhoea | Multiple pathogens | Culture | PCR | 432 | 41 (9.5%) | 14 (3.2%) | 7 |  |
| Njuguna et al. (2013)^47^ | Surveillance | Nairobi county | Rural community | Community children with diarrhoea | *Shigella* | Culture |  | 2476 | 389 (16%) |  | 7 |  |
| Karambu S. et al. (2014)^48^ | Cross-sectional | Meru county | Urban health facility | OCD | Multiple pathogens | Culture | PCR | 308 | 9 (3%) | 30 (10%) | 7 |  |
| Nyanga PL. et al. (2017)^49^ | Cross-sectional | Nairobi county | Urban health facility | OCD | *Shigella* and *E. coli* | Culture | PCR | 354 | 14 (4%) | 37 (10.5%) | 5 |  |
| Mbuthia OW. et al. (2018)^50^ | Cross-sectional | Murang’a county | Urban and rural health facilities | OCD | Multiple pathogens | Culture | PCR | 163 | 14 (9%) | 8 (5%) | 6 |  |
| Schilling et al. (2017)^51^ | Surveillance | Siaya county | Rural health facilities | OCD | Multiple pathogens | Culture | PCR | 1020 | 78 (8%) | 148 (15%) | 8 |  |
| O'Reilly et al. (2012)^52^ | Surveillance | Siayi county | Rural health facility | Hospitalised children with diarrhoea | Multiple pathogens | Culture |  | 1137 | 42 (4%) |  | 6 |  |
| Shah M et al. (2017)^53^ | Surveillance | Kiambu and Homabay counties | Urban and rural health facilities | Hospitalised children with diarrhoea | Multiple pathogens | Culture | PCR | 1060 | 14 (1%) | 113 (11%) | 6 |  |
| Boru et al. (2013)^54^ | Case-control | Nairobi county | Urban health facility | Outpatient children with and without diarrhoea | Multiple pathogens | Culture | PCR | 41 | 2 (5%) | 3 (7%) | 6 |  |
| Webale et al. (2020)^55^ | Cross-sectional | Nairobi county | Urban health facility | OCD | Multiple pathogens | Culture | PCR | 374 | 12 (3%) | 38 (10%) | 5 |  |
| Makobe et al. (2012)^56^ | Surveillance | Nairobi county | Urban health facility | Hospitalised children with diarrhoea | *E. coli* | NA | PCR | 207 | NA | 15 (7%) | 3 |  |
| Bii et al. (2005)^57^ | Cross-sectional | Nairobi county | Urban health facility | OCD | *E. coli* | NA | PCR | 82 | NA | 22 (27%) | 3 |  |
| Kipkirui et al. (2021)^58^ | Surveillance | Kissi county | Rural health facility | OCD | ETEC | NA | PCR | 225 | NA | 23 (10.2%) | 6 |  |
| **Malawi** | | | | | | | | | | | | |
| Ndungo E. et al. (2022)^59^ | Surveillance | Chikhwawa District | Rural community | Community children with diarrhoea | *Shigella* | PCR | NA | 369 | 37 (10%) | NA | 8 |  |
| Iturriza-Gómara M. et al. (2019)^60^ | Case-control | Blantyre district | Urban health facility | Hospitalised children with diarrhoea and community controls | Multiple pathogens | PCR | PCR | 684 | 108 (16%) | 213 (31%) | 7 |  |
| Versloot et al. (2018)^61^ | Cross-sectional | Blantyre district | Urban health facility | Hospitalised children with complicated SAM | Multiple pathogens | PCR | PCR | 47 | 19 (40%) | 10 (21%) | 5 |  |
| Attia et al. (2016)^62^ | Cross-sectional | Blantyre district | Urban health facility | Hospitalised children with complicated SAM | Multiple pathogens | PCR | PCR | 64 | 23 (35%) | 10 (16%) | 7 |  |

OCD: Outpatient Children with Diarrhoea; SAM: Severe Acute Malnutrition; PCR: Polymerase Chain Reaction; NA: Not Applicable; SNNP: Southern Nations, Nationalities and Peoples

*Note: The sample size refers to persons or number individuals tested

**Table 2: Risk of bias assessment for shigella and ETEC studies**

|  |  |  | **Selection** | | | | **Comparability** | **Outcome** | | | |
| --- | --- | --- | --- | --- | --- | --- | --- | --- | --- | --- | --- |
| **Ref.** | **Country** | **Study type** | **Representativeness of the sample** | **Sample size** | **Non-respondents** | **Ascertainment of exposure** | **Based on design and analysis** | **Assessment of outcome** | **Statistical test** | | **Total score** |
| Debas G. et al. (2011) | Ethiopia | Cross-sectional | * |  |  | * | * | ** | * | | 6 |
| Abebe W. et al. (2018) | Ethiopia | Cross-sectional | * | * |  | * | * | ** | * | | 7 |
| Dessale M. et al. (2023) | Ethiopia | Cross-sectional | * | * |  | * | * | ** | * | | 7 |
| Balew M. et al. (2023) | Ethiopia | Cross-sectional | * | * |  | * | * | ** | * | | 7 |
| Kahsay AG. et al. (2015) | Ethiopia | Cross-sectional | * | * |  | * | * | ** | * | | 7 |
| Getamesay M. et al. (2014) | Ethiopia | Cross-sectional | * |  |  | * | * | ** | * | | 6 |
| Abera B. et al. (2020) | Ethiopia | Cross-sectional | * | * |  | * | * | ** | * | | 7 |
| Ameya G. et al. (2018) | Ethiopia | Cross-sectional | * |  |  | * | * | ** | * | | 6 |
| Feleke H. et al. (2018) | Ethiopia | Cross-sectional | * | * |  | * | * | ** | * | | 7 |
| Zelelia T. et al. (2019) | Ethiopia | Cross-sectional | * | * |  | * | * | ** | * | | 7 |
| Gebreegziabher G. et al. (2018) | Ethiopia | Cross-sectional | * | * |  | * | * | ** | * | | 7 |
| Tosisa et al. (2020) | Ethiopia | Cross-sectional | * | * |  | * | * | ** | * | | 7 |
| Beyene et al. (2014) | Ethiopia | Cross-sectional | * | * |  | * | * | ** |  | | 6 |
| Ayele B. et al. (2023) | Ethiopia | Cross-sectional | * | * |  | * | * | ** | * | | 7 |
| Assefa et al. (2019) | Ethiopia | Cross-sectional | * | * |  | * | * | ** | * | | 7 |
| Admassu et al. (2015) | Ethiopia | Cross-sectional | * | * |  | * | * | ** |  | | 6 |
| Mamuye et al. (2015) | Ethiopia | Cross-sectional | * | * |  | * | * | ** | * | | 7 |
| Mekonnen et al. (2018) | Ethiopia | Observational | * | * |  | * | * | ** | * | | 7 |
| Mulu W. et al. (2017) | Ethiopia | Surveillance | * | * |  | * | * | ** | * | | 7 |
| Leting et al. (2021) | Kenya | Cross-sectional | * | * |  | * |  | ** |  | | 5 |
| Zachariah et al. (2021) | Kenya | Cross-sectional | * | * |  | * | * | ** | * | | 7 |
| Karambu S. et al. (2013) | Kenya | Cross-sectional | * |  |  | * | ** | ** | * | | 7 |
| Nyanga PL. et al. (2017) | Kenya | Cross-sectional | * |  |  | * | * | ** |  | | 5 |
| Mbuthia OW. et al. (2018) | Kenya | Cross-sectional | * | * |  | * | ** | * |  | | 6 |
| Webale et al. (2020) | Kenya | Cross-sectional | * |  |  | * | * | ** |  | | 5 |
| Sang et al. (2012) | Kenya | Cross-sectional | * |  |  | * | * | ** | * | | 6 |
| Shah M et al. (2017) | Kenya | Surveillance | * |  |  | * | * | ** | * | | 6 |
| Pavlinac et al. (2015) | Kenya | Surveillance | * | * |  | * | ** | ** | * | | 8 |
| Beatty et al. (2009) | Kenya | Surveillance | * | * |  | * | * | ** | * | | 7 |
| Njuguna et al. (2013) | Kenya | Surveillance | * | * |  | * | * | ** | * | | 7 |
| Schilling et al. (2017) | Kenya | Surveillance | * | * |  | * | ** | ** | * | | 7 |
| O'Reilly et al. (2012) | Kenya | Surveillance | * |  |  | * | * | ** | * | | 6 |
| Versloot et al. (2018) | Malawi | Cross-sectional | * |  |  | * | * | ** | * | | 6 |
| Attia et al. (2016) | Malawi | Cross-sectional |  |  |  | * | ** | ** | * | | 6 |
| Ndungo E. et al. (2022) | Malawi | Surveillance | * | * | * | * | * | ** | * | | 8 |
| Makobe et al. (2012) | Kenya | Surveillance | * |  |  | * |  | * |  | | 3 |
| Bii et al. (2005) | Kenya | Cross-sectional |  |  |  | * |  | ** |  | | 3 |
| Kipkirui et al. (2021) | Kenya | Cross-sectional | * | * |  | * |  | ** | * | | 6 |
|  |  |  | **Selection** | | | | **Comparability** | **Exposure** | | | |
| **Ref.** | **Country** | **Study type** | **Is the case definition adequate?** | **Representativeness of the cases** | **Selection of Controls** | **Definition of Controls** | **Based on design and analysis** | **Ascertainment of exposure** | **Same method of ascertainment for cases and controls** | **Non-Response rate** | **Total score** |
| Mekonnen GK. et al. (2019) | Ethiopia | Case-control | * | * | * | * | * | * | * |  | 7 |
| Kasumba et al. (2023) | Kenya | Case-control | * | * | * | * | * | * | * |  | 7 |
| Boru et al. (2013) | Kenya | Case-control | * | * |  | * | * | * | * |  | 6 |
| Swierczewski BE. et al. (2013) | Kenya | Case-control | * |  | * | * | * |  | * | * | 6 |
| Iturriza-Gómara M. et al. (2019) | Malawi | Case-control | * | * | * | * | ** | * |  |  | 7 |

**Table 3: Shigella strain isolates**

| **Ref.** | **Shigella isolate tested** | **Shigella sonnei** | **Shigella flexneri** | **Shigella boydii** | **Shigella dysenteriae** |
| --- | --- | --- | --- | --- | --- |
| **Ethiopia** | | | | | |
| Gebreegziabher G. et al 2018 | 18 | 2 | Not indicated | Not indicated | Not indicated |
| Dessale et al 2023 | 19 | Not indicated | Not indicated | Not indicated | 8 |
| Getamesay M et al 2015 | 11 | 0 | 11 | 0 | 0 |
| Tosisa et al 2020 | 6 | 1 | 3 | 2 | 0 |
| Admassu et al 2015 | 40 | 0 | 18 | 10 | 12 |
| **Kenya** | | | | | |
| Leting et al 2021 | 18 | 3 | 7 | 4 | 4 |
| Pavlinac et al 2015 | 63 | 28 | 21 | 1 | 2 |
| Kasumba et al 2023 | 128 | 35 | 81 | 10 | 2 |
| Beatty et al 2009 | 116 | 23 | 71 | 9 | 9 |
| Swierczewski BE. et al. 2012 | 41 | 13 | 16 | Not indicated | 3 |
| Nyanga PL et al 2017 | 14 | 3 | 6 | 3 | 2 |
| Karambu et al 2013 | 9 | 0 | 6 | 0 | 3 |
| Mbuthia O et al 2018 | 14 | 6 | 0 | 8 | 0 |
| Boru et al 2013 | 2 | 0 | 1 | 0 | 1 |
| Webale et al 2020 | 12 | 2 | 7 | 2 | 1 |
| **TOTAL** | **511** | **116** | **248** | **49** | **47** |

**Table 4: ETEC serotype strains**

| **Ref.** | **ETEC isolates tested** | **Strain with both LT and ST** | **Strain with LT** | **Strain with ST** |
| --- | --- | --- | --- | --- |
| **Kenya** | | | | |
| Kipkirui E., et al 2021 | 23 | 2 | 5 | 16 |
| Sang WK et al 2012 | 8 | 5 | 2 | 1 |
| Mbuthia et al 2018 | 8 | 8 | 0 | 0 |
| Nyanga et al 2017 | 37 | 23 | 8 | 6 |
| Shah et al 2017 | 113 | 13 | 37 | 63 |
| Schilling et al 2017 | 148 | 0 | 56 | 92 |
| Bii et al 2005 | 12 | 5 | 4 | 3 |
| **Malawi** | | | | |
| Iturriza-Gómara et al 2019 | 213 | 0 | 68 | 145 |
| **TOTAL** | **562** | **56** | **180** | **326** |

HT: Heal-labile toxin; ST: Heat-stable toxin

**Table 5: Antibiotic resistance N (%) for shigella in Ethiopia and Kenya**

|  |  | **Ethiopia** | | | | | | | | | | | | | | | | **Kenya** | | | | | | | | |
| --- | --- | --- | --- | --- | --- | --- | --- | --- | --- | --- | --- | --- | --- | --- | --- | --- | --- | --- | --- | --- | --- | --- | --- | --- | --- | --- |
|  |  | **Abera B. et al 2020** | **Ameya G et al 2018** | **Feleke H. et al 2018** | **Zelelia Tizazu. et al 2019** | **Mekonnen GK. Et al 2019** | **Gebreegziabher G. et al 2018** | **Abebe W et al 2018** | **Dessale et al 2023** | **Getamesay et al 2014** | **Tosisa et al 2020** | **Mekonnen M et al 2018** | **Beyene et al 2014** | **Mamuye et al 2015** | **Ayele B et al 2023** | **Assefa et al 2019** | **Admassu et al 2015** | **Swierczewski BE. et al. 2012** | **Nyanga PL et al 2017** | **Leting et al 2021** | **Pavlinac et al 2015** | **Zachariar et al 2021** | **Sang et al 2012** | **Kasumba et al 2023** | **Webale et al 2020** | **Beatty et al 2009** |
| **Classification** | **Antibiotics** | **4 (100)** | **8 (100)** | **5 (100)** | **3 (100)** | **16 (100)** | **18 (100)** | **17 (100)** | **19 (100)** | **11 (100)** | **6 (100)** | **11 (100)** | **6 (100)** | **23 (100)** | **47 (100)** | **18 (100)** | **40 (100)** | **45 (100)** | **14 (100)** | **18 (100)** | **63 (100)** | **28 (100)** | **15 (100)** | **160 (100)** | **21 (100)** | **116 (100)** |
| **Penicillins** | Amoxicillin | **1 (25)** | **8 (100)** | **5 (100)** | **0 (0)** | **16 (100)** | NA | NA | **15 (79)** | **11 (100)** | **3 (50)** | **11 (100)** | **6 (100)** | NA | **47 (100)** | **18 (100)** | **26 (65)** | NA | **1 (7)** | **16 (89)** | NA | NA | NA | NA | **4 (19)** | **70 (60)** |
|  | Ampicillin | NA | NA | **5 (100)** | **1 (33)** | **16 (100)** | **16 (89)** | **14 (82)** | **14 (74)** | **7 (64)** | **6 (100)** | **10 (91)** | **6 (100)** | **22 (96)** | **44 (94)** | NA | **36 (90)** | **21 (47)** | **12 (86)** | **18 (100)** | **30 (48)** | **23 (82)** | **9 (60)** | **94 (59)** | **13 (29)** | **79 (68)** |
|  | Augmentin | NA | NA | NA | NA | NA | NA | NA | NA | NA | NA | NA | NA | **21 (91)** | NA | NA | NA | NA | NA | NA | NA | NA | NA | NA | NA | NA |
| **Cephalosporins** | Ceftriaxone | **1 (25)** | NA | **0 (0)** | **0 (0)** | NA | **0 (0)** | **3 (18)** | **1 (5)** | **6 (55)** | NA | **0 (0)** | NA | **1 (43)** | NA | **0 (0**) | NA | NA | **2 (14)** | **1 (6)** | NA | **9 (32)** | NA | **0 (0)** | **10 (48)** | **0 (0)** |
|  | Cefuroxime | NA | NA | NA | NA | NA | NA | NA | NA | NA | NA | NA | NA | NA | NA | NA | NA | NA | NA | NA | NA | **15 (54)** | NA | NA | NA | NA |
|  | Cephalexine | **1 (25)** | NA | NA | NA | NA | NA | NA | NA | NA | NA | NA | NA | NA | NA | NA | NA | NA | NA | NA | NA | NA | NA | NA | NA | NA |
|  | Cefotaxime | NA | NA | NA | NA | NA | NA | NA | NA | NA | **0 (0)** | NA | NA | NA | NA | NA | NA | NA | NA | NA | NA | NA | NA | NA | NA | NA |
|  | Ceftizoxime | NA | NA | NA | NA | NA | NA | NA | NA | NA | NA | NA | NA | NA | NA | NA | **11 (28)** | NA | NA | NA | NA | NA | NA | NA | NA | NA |
|  | Cefoxitin | NA | NA | NA | NA | NA | NA | NA | NA | NA | NA | NA | NA | NA | **10 (21)** | NA | NA | NA | NA | NA | NA | NA | NA | NA | NA | NA |
|  | Cephalotin | NA | NA | NA | **0 (0)** | NA | NA | NA | NA | **0 (0)** | NA | NA | NA | NA | NA | NA | NA | NA | NA | NA | NA | NA | NA | NA | NA | NA |
|  | Ceftazidime | NA | NA | NA | NA | **0 (0)** | NA | NA | **3 (16)** | NA | NA | NA | NA | NA | NA | NA | NA | NA | NA | **3 (17)** | NA | NA | NA | NA | NA | NA |
| **Quinolones & Flouroquinolones** | Norfloxacin | NA | **1 (13)** | NA | NA | NA | **0 (0)** | **0 (0)** | NA | NA | NA | NA | NA | NA | **0 (0)** | NA | **5 (13)** | NA | NA | NA | NA | **10 (36)** | **0 (0)** | NA | NA | NA |
|  | Ciprofloxacin | NA | **0 (0)** | NA | **0 (0)** | NA | **0 (0)** | **3 (18)** | **0 (0)** | **0 (0)** | **5 (83)** | **1 (9)** | NA | **1 (43)** | **6 (13)** | **0 (0)** | **3 (8)** | **2 (4)** | **2 (14)** | NA | **0 (0)** | **8 (29)** | **1 (7)** | NA | **4 (19)** | **0 (0)** |
|  | Nalidixic acid | NA | NA | NA | **0 (0)** | NA | **5 (28)** | **0 (0)** | NA | **0 (0)** | **5 (83)** | **2 (18)** | **1 (9)** | **5 (22)** | **3 (6)** | NA | NA | NA | **2 (14)** | NA | NA | **11 (39)** | **1 (7)** | **2 (1)** | **4 (19)** | **3 (3)** |
| **Aminoglycosides** | Kanamycin | NA | NA | NA | NA | **0 (0)** | NA | **0 (0)** | NA | NA | NA | NA | NA | NA | NA | NA | NA | NA | **0 (0)** | NA | NA | NA | NA | NA | **0 (0)** | **0 (0)** |
|  | Amikacin | NA | NA | NA | NA | NA | NA | NA | NA | NA | **0 (0)** | NA | NA | NA | NA | NA | NA | NA | NA | **1 (6)** | NA | NA | NA | NA | NA | NA |
|  | Streptomycin | NA | NA | NA | NA | NA | NA | NA | NA | NA | NA | NA | NA | NA | NA | NA | NA | NA | **13 (93)** | NA | NA | NA | NA | NA | **12 (57)** | **111 (96)** |
|  | Gentamicin | NA | **2 (25)** | **3 (60)** | **0 (0)** | NA | **5 (28)** | **13 (77)** | NA | **3 (27)** | **0 (0)** | **3 (27)** | NA | **4 (17)** | **10 (21)** | NA | **4 (10)** | NA | **0 (0)** | **5 (28)** | NA | **4 (14)** | **0 (0)** | NA | **2 (10)** | **1 (0.1)** |
| **Macrolides** | Erythromycin | NA | **5 (63)** | NA | NA | **9 (56)** | NA | NA | NA | **10 (91)** | NA | NA | NA | NA | **47 (100)** | NA | NA | NA | **0 (0)** | NA | NA | **26 (93)** | NA | NA | **2 (10)** | NA |
|  | Azithromycin | NA | NA | NA | NA | NA | NA | NA | **8 (42)** | NA | NA | NA | NA | NA | NA | NA | NA | NA | NA | NA | NA | NA | NA | **1 (0.6)** | NA | NA |
| **Tetracyclins** | Tetracyclin | NA | NA | **3 (60)** | **2 (67)** | **7 (44)** | **14 (78)** | NA | **13 (68)** | **6 (55)** | **4 (67)** | NA | NA | NA | **43 (91)** | **18 (100)** | **24 (60)** | **32 (71)** | **12 (86)** | NA | **47 (75)** | NA | **9 (60)** | NA | **14 (67)** | **109 (94)** |
|  | Doxycycline | NA | NA | NA | NA | NA | NA | NA | **10 (53)** | NA | NA | NA | NA | NA | **39 (83)** | **16 (89)** | NA | NA | NA | NA | NA | **23 (82)** | NA | NA | NA | NA |
|  | Minocycline | NA | NA | NA | NA | NA | NA | NA | NA | NA | NA | NA | NA | NA | NA | NA | NA | NA | NA | NA | NA | **19 (68)** | NA | NA | NA | NA |
| **Chloramphenicol** | Chloramphenicol | NA | **4 (50)** | **2 (40)** | **1 (33)** | **8 (50)** | **10 (65)** | **8 (47)** | **3 (16)** | **1 (9)** | **3 (50)** | **5 (46)** | **1 (17)** | **5 (22)** | **32 (68)** | **18 (100)** | **14 (35)** | NA | **8 (57)** | NA | NA | NA | **5 (33)** | NA | **12 (57)** | **66 (57)** |
| **Lincosamides** | Clindamycin | NA | **3 38)** | NA | NA | NA | NA | NA | NA | NA | NA | NA | NA | NA | NA | NA | NA | NA | NA | NA | NA | NA | NA | NA | NA | NA |
| **Sulfonamides** | Trimethoprim-Sulphamethoxazole | **3(75)** | **2(25)** | **2(40)** | **1 (33)** | **4(25)** | **10(65)** | **11 (65)** | **11 (58)** | **0(0)** | **3(50)** | **8(73)** | **6(100)** | **12 (52)** | **27(57)** | NA | **19 (48)** | **40 (89)** | **8 (57)** | **6 (33)** | **57(91)** | **20(71)** | **3 (20)** | **157 (98)** | **10 (48)** | **113 (97)** |
|  | Sulfisoxazole | NA | NA | NA | NA | NA | NA | NA | NA | NA | NA | NA | NA | NA | NA | NA | NA | NA | NA | NA | NA | NA | NA | NA | NA | **111 (96)** |
|  | MDR | - | - | - | - | - | - | - | **16 (84)** | **11 (100)** | - | - | - | - | **40 (85)** | **18 (100)** | - | - | - | - | **22 (35)** | - | - | - | - | - |

NA: Not Applicable (The antibiotic was not tested); MDR: Multi-Drug Resistant
