# Supplementary material for: Burden of Shigella and enterotoxigenic Escherichia coli infections among children under 5 years in Ethiopia, Kenya and Malawi: a systematic review and meta-analysis
Source: BMJ Glob Health. 2026 Mar 2;11(3):e018515. doi: 10.1136/bmjgh-2024-018515 (PMC12958989; doi:10.1136/bmjgh-2024-018515)
Supplement: online supplemental file 4 [file bmjgh-11-3-s004.pdf]

Supplementary appendix 4

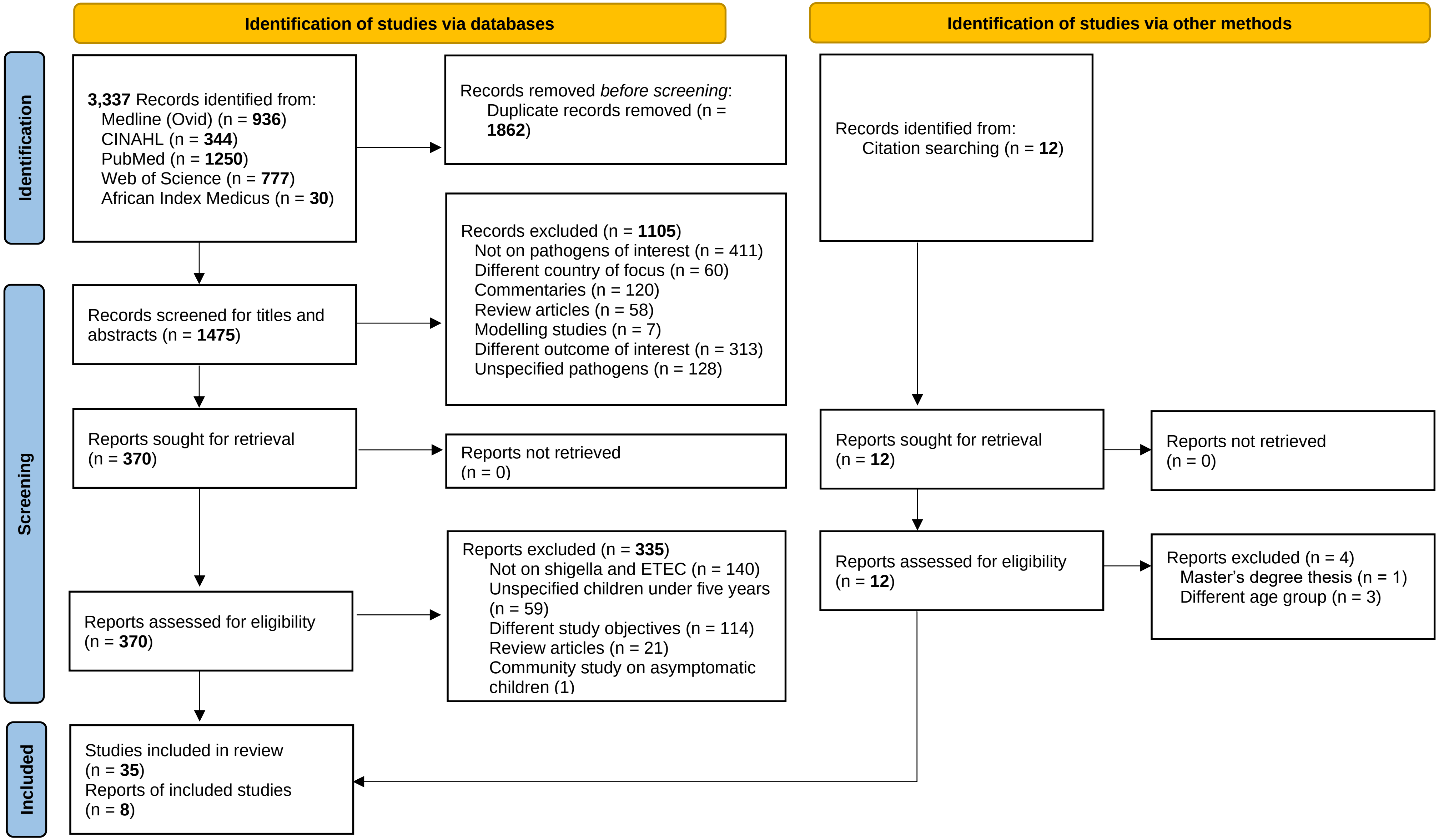

Figure 1: Study selection process from online datasets and cross-referencing

From: Page MJ, McKenzie JE, Bossuyt PM, Boutron I, Hoffmann TC, Mulrow CD, et al. The PRISMA 2020 statement: an updated guideline for reporting systematic reviews. BMJ 2021;372:n71. doi: 10.1136/bmj.n71.

For more information, visit: http://www.prisma-statement.org/

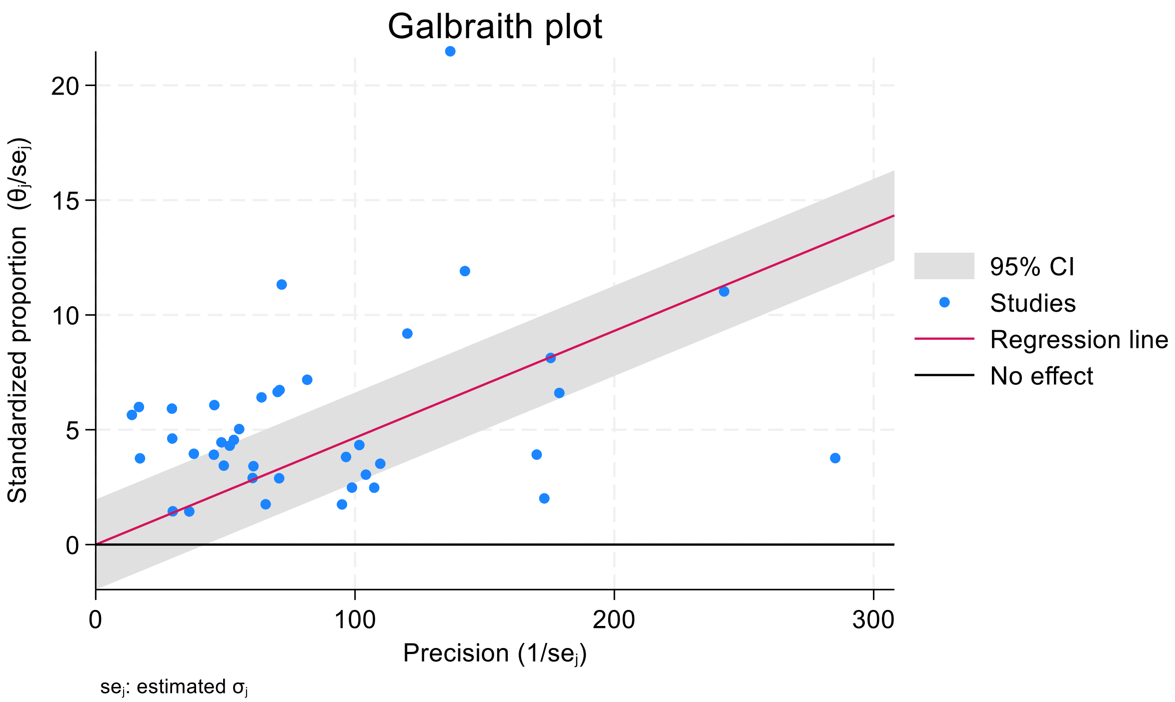

Figure 2: Galbraith plot of proportion*precision / standard error against precision (1/standard error) for *Shigella* prevalence among U5 children in Ethiopia, Kenya and Malawi

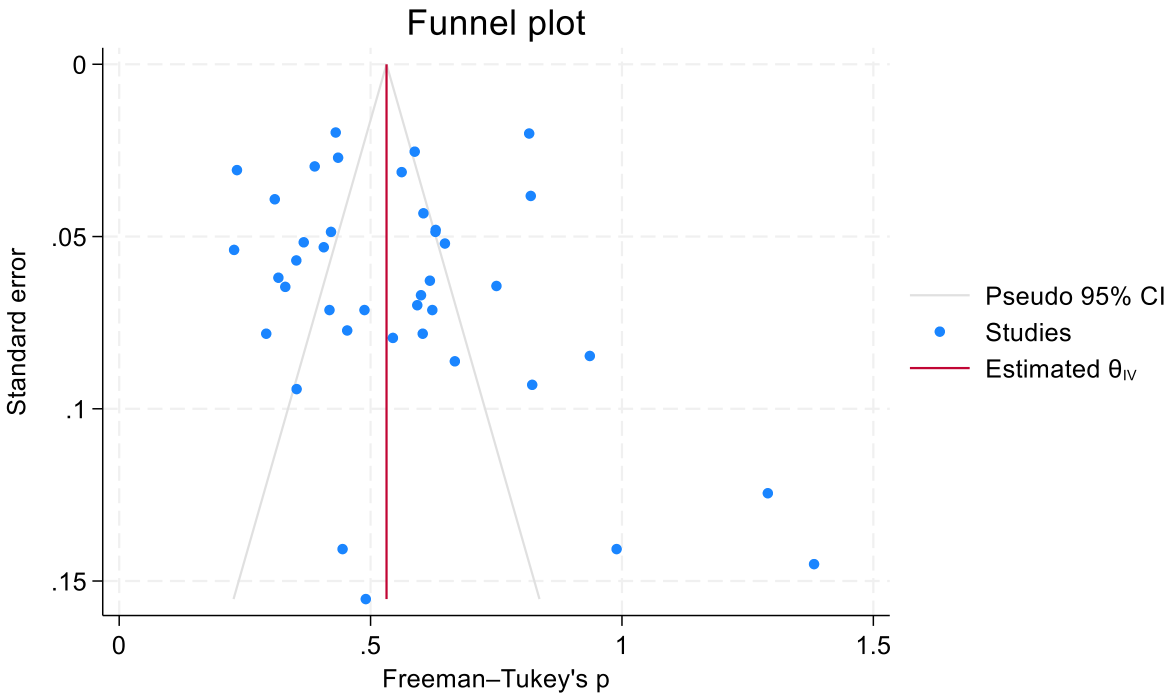

Figure 3: Freeman-Turkey transformed funnel plot for *Shigella* studies in the meta-analysis

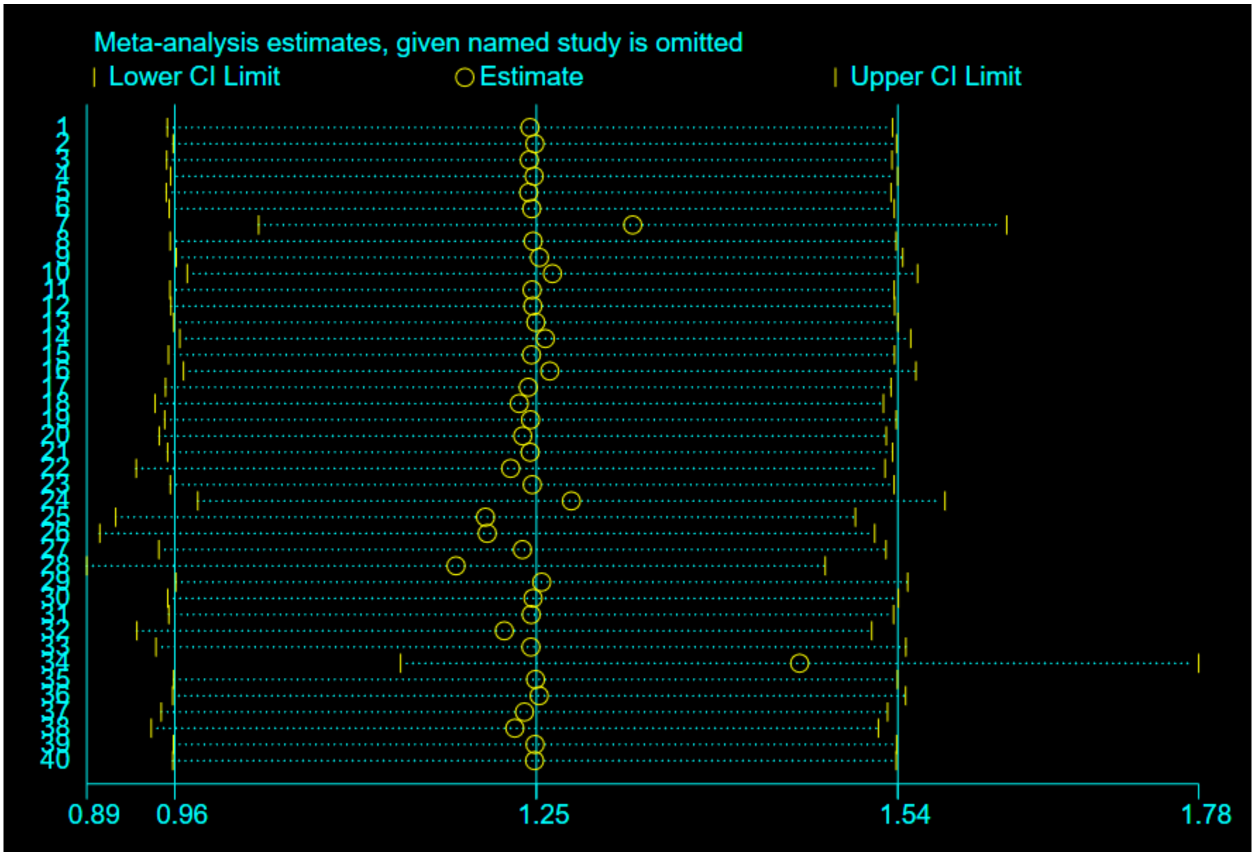

Figure 4: Sensitivity analysis for *Shigella* studies in the meta-analysis

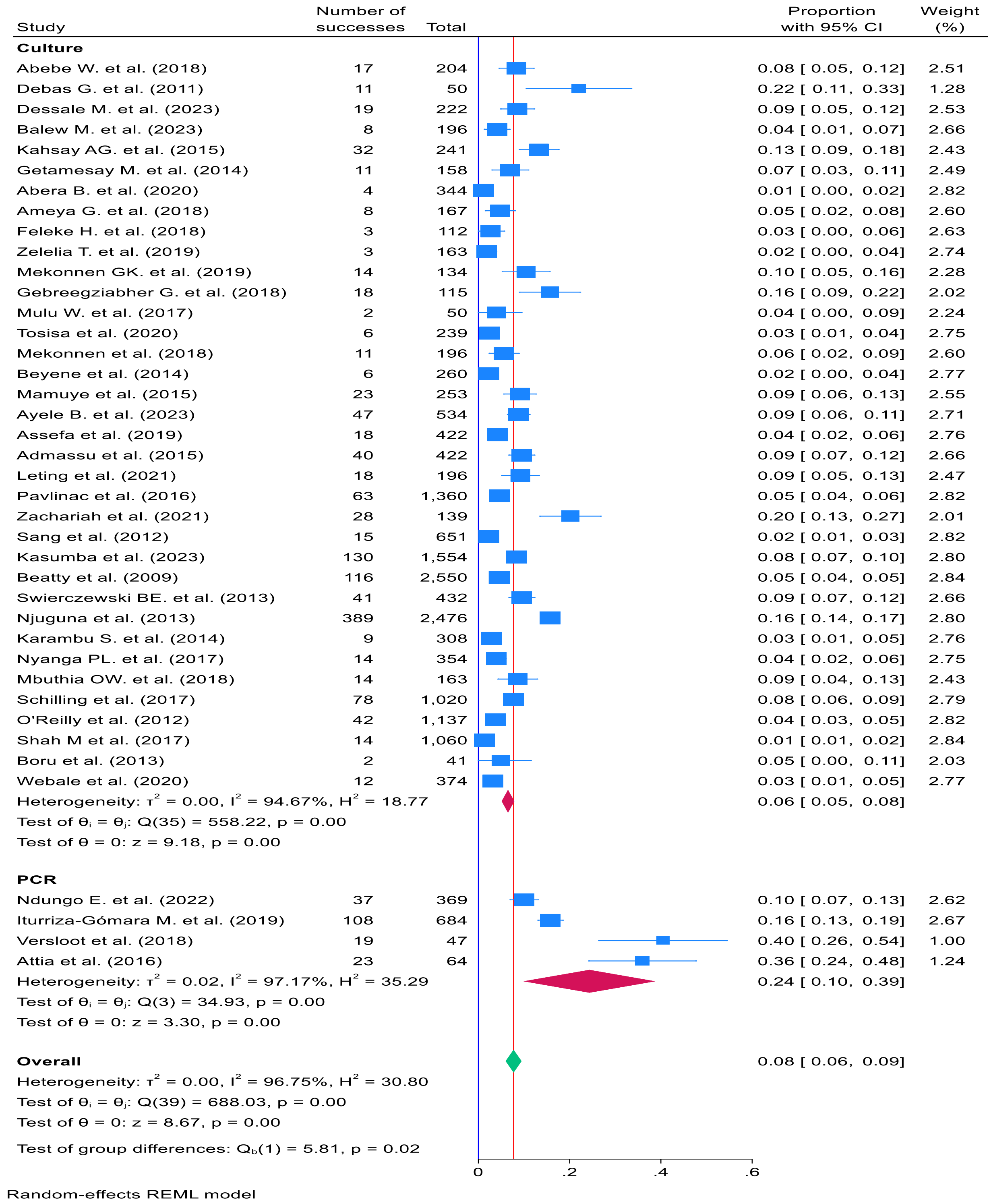

Figure 5: Forest plot of the subgroup pooled prevalence by pathogen identification of *Shigella* infection among under five children in Ethiopia, Kenya and Malawi

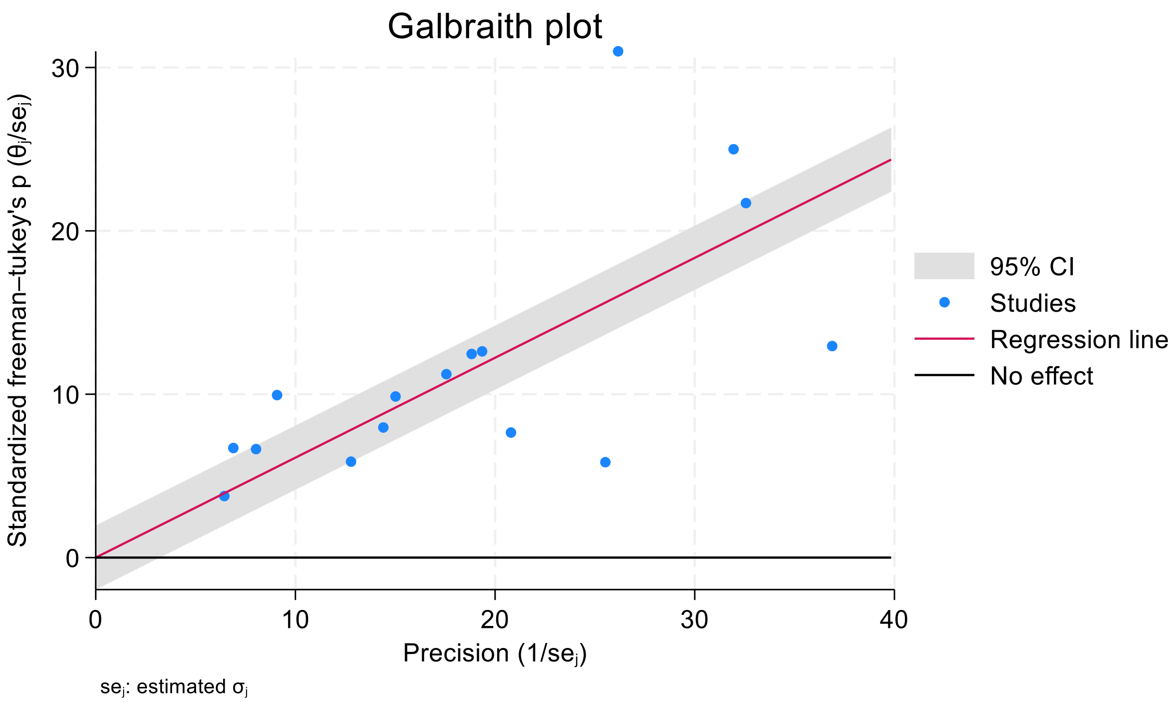

Figure 6: Galbraith plot of proportion*precision / standard error against precision (1/standard error) for ETEC prevalence among U5 children in Kenya and Malawi

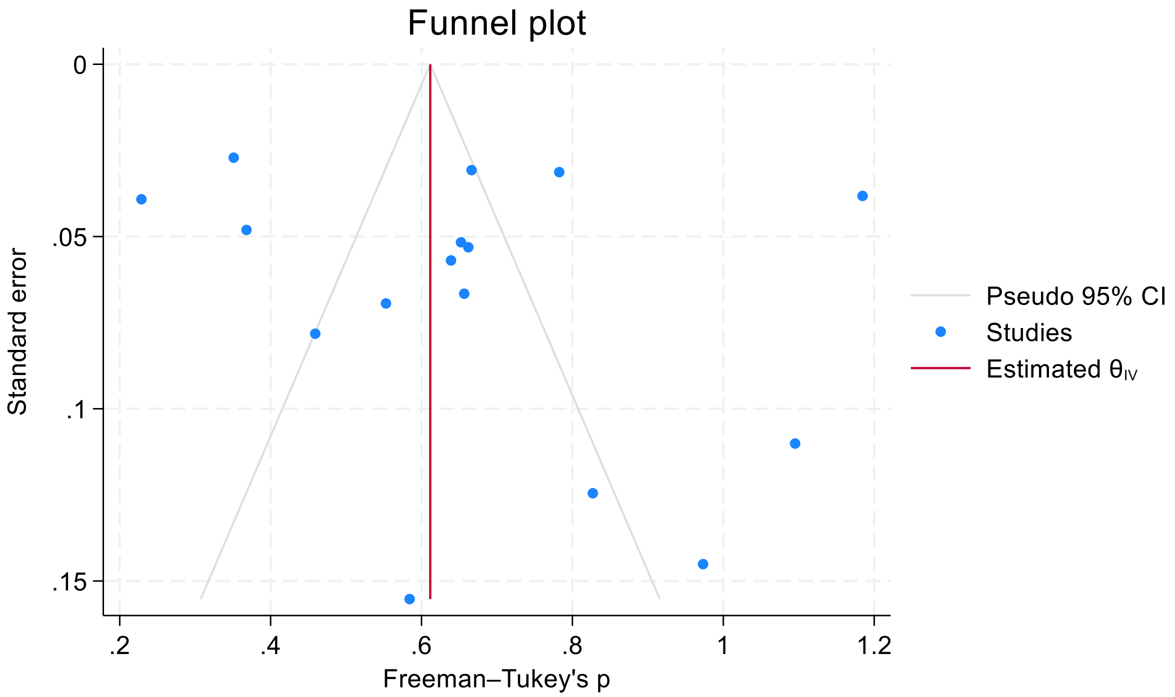

Figure 7: Freeman-Turkey transformed funnel plot for ETEC studies in the meta-analysis

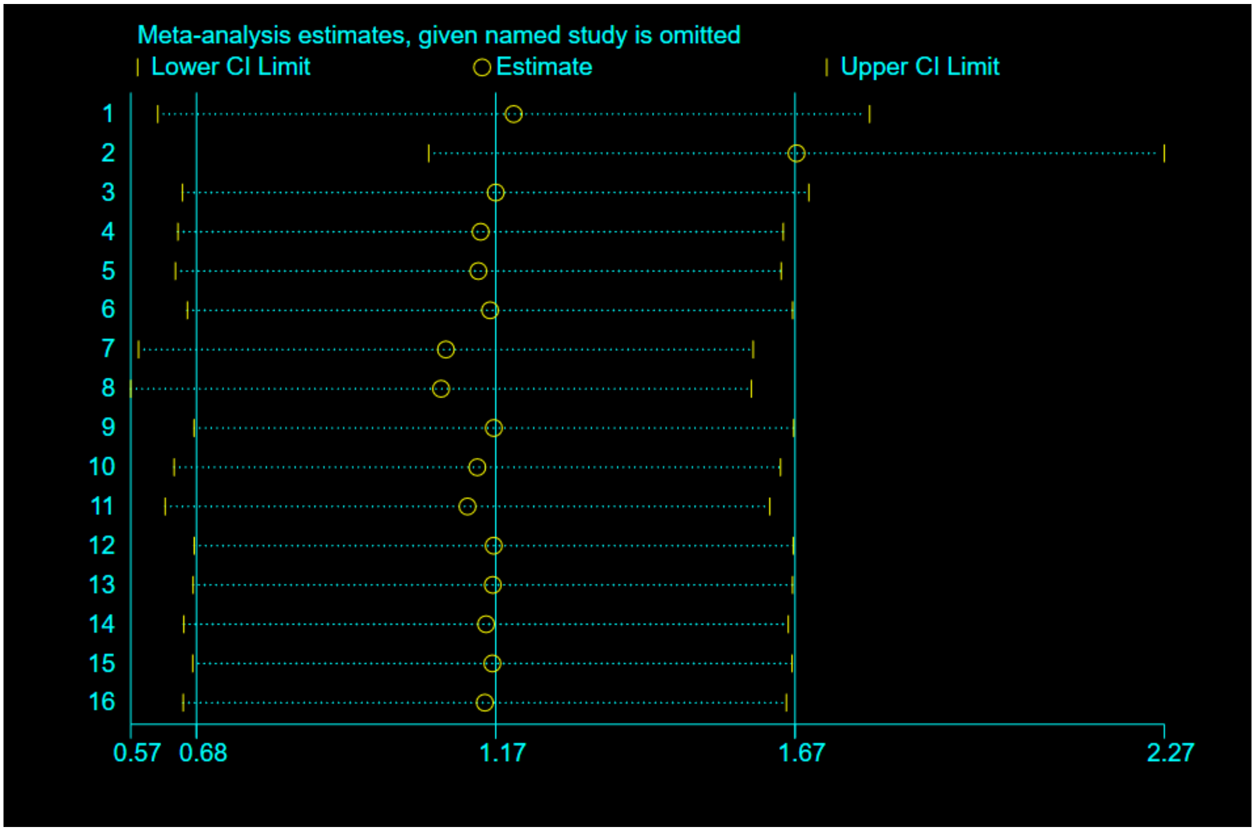

Figure 8: Sensitivity analysis for ETEC studies in the meta-analysis
